# Tumour-reactive heterotypic CD8 T cell clusters from clinical samples

**DOI:** 10.1038/s41586-025-09754-w

**Published:** 2025-11-19

**Authors:** Sofía Ibáñez-Molero, Johanna Veldman, Juan Simon Nieto, Joleen J. H. Traets, Austin George, Kelly Hoefakker, Anita Karomi, Rolf Harkes, Bram van den Broek, Su Min Pack, Liselotte Tas, Nils L. Visser, Susan E. van Hal-van Veen, Paula Alóndiga-Mérida, Maartje Alkemade, Iris M. Seignette, Renaud Tissier, Marja Nieuwland, Martijn van Baalen, Joanna Poźniak, Erik Mul, Simon Tol, Sofia Stenqvist, Lisa M. Nilsson, Jonas A. Nilsson, John B. A. G. Haanen, Winan J. van Houdt, Daniel S. Peeper

**Affiliations:** 1 Division of Molecular Oncology and Immunology, Oncode Institute, Netherlands Cancer Institute, Amsterdam, The Netherlands; 2 Division of Tumor Biology and Immunology, Netherlands Cancer Institute, Amsterdam, The Netherlands; 3 BioImaging Facility, Netherlands Cancer Institute, Amsterdam, The Netherlands; 4 Core Facility Molecular Pathology & Biobanking, Netherlands Cancer Institute, Amsterdam, The Netherlands; 5 Department of Pathology, Netherlands Cancer Institute, Amsterdam, The Netherlands; 6 Biostatistics Unit, Netherlands Cancer Institute, Amsterdam, The Netherlands; 7 Genomic Core Facility, Netherlands Cancer Institute, Amsterdam, The Netherlands; 8 Flow Cytometry Facility, Netherlands Cancer Institute, Amsterdam, The Netherlands; 9 Laboratory for Molecular Cancer Biology, Department of Oncology, VIB Center for Cancer Biology, KU Leuven, Leuven, Belgium; 10 Flow Cytometry Facility, Sanquin, Amsterdam, The Netherlands; 11 Sahlgrenska Center for Cancer Research, Institute of Clinical Sciences, Sahlgrenska Academy, University of Gothenburg, Gothenburg, Sweden; 12 Harry Perkins Institute of Medical Research and University of Western Australia, Perth, Western Australia Australia; 13 Department of Medical Oncology, Netherlands Cancer Institute, Amsterdam, The Netherlands

**Keywords:** Cancer microenvironment, Tumour immunology

## Abstract

Emerging evidence suggests a correlation between CD8^+^ T cell–tumour cell proximity and anti-tumour immune response^1,2^. However, it remains unclear whether these cells exist as functional clusters that can be isolated from clinical samples. Here, using conventional and imaging flow cytometry, we show that from 21 out of 21 human melanoma metastases, we could isolate heterotypic clusters, comprising CD8^+^ T cells interacting with one or more tumour cells and/or antigen-presenting cells (APCs). Single-cell RNA-sequencing analysis revealed that T cells from clusters were enriched for gene signatures associated with tumour reactivity and exhaustion. Clustered T cells exhibited increased TCR clonality indicative of expansion, whereas TCR-matched T cells showed more exhaustion and co-modulation when conjugated to APCs than when conjugated to tumour cells. T cells that were expanded from clusters ex vivo exerted on average ninefold increased killing activity towards autologous melanomas, which was accompanied by enhanced cytokine production. After adoptive cell transfer into mice, T cells from clusters showed improved patient-derived melanoma control, which was associated with increased T cell infiltration and activation. Together, these results demonstrate that tumour-reactive CD8^+^ T cells are enriched in functional clusters with tumour cells and/or APCs and that they can be isolated and expanded from clinical samples. Typically excluded by single-cell gating in flow cytometry, these distinct heterotypic T cell clusters are a valuable source to decipher functional tumour–immune cell interactions and may also be therapeutically explored.

## Abstract (web summary)

Tumour-reactive CD8^+^ T cells are enriched in functional clusters with tumour cells and/or antigen-presenting cells and can be isolated and expanded from clinical samples.

## Main

An increasing body of evidence suggests that, in addition to the type, density and state of immune cells in the tumour microenvironment (TME), their proximity to cancer cells also influences immunotherapy outcomes^1–5^. For example, in two melanoma studies, favourable responses to immune checkpoint inhibitors are associated with either higher densities of CD8^+^ tumour-infiltrating lymphocytes (TILs) within a distance of 20 μm of melanoma cells^5^ or a higher proportion and closer proximity of (proliferating) antigen-experienced CD8^+^ T cells to the tumour cells^6^. Similarly, upon anti-PD-1 and chemoradiotherapy, a higher proportion of on-treatment PD-1^+^CD4^+^ and CD8^+^ T cells within 100 μm of tumour cells predicts longer overall survival in oesophageal cancer^7^, while, in locally advanced cervical cancer, progression-free patients show closer proximity of CD3^+^ TILs to PD-L1^+^ tumour cells^8^. Furthermore, an automated image classifier characterizing interactions between TILs and non-TILs can predict immunotherapy outcome^9^.

These notions are consistent with the understanding that, after specific antigen recognition, cytotoxic T cells physically engage their target cells through their TCRs, followed by immunological synapse formation^10^. The structural and functional avidity of cytotoxic CD8^+^ T cells are important parameters for infiltration into and activity against tumours^11^. It takes successive interactions and dynamic contacts for T cells to effectively eliminate cancer cells^12,13^. The importance of direct interactions between cytotoxic T cells and tumour cells has been confirmed by single-cell sequencing analyses, which have been instrumental for our understanding of TME complexity^14–19^. Additional information can be extracted when cells are isolated if clustered with neighbouring cells^14,16–18,20^. For example, relative to unconjugated tumour cells, mouse circulating tumour cells (CTCs) associated with neutrophils show increased cell cycle activity and metastatic potential^21^. Physically interacting cells have also been analysed by integrated single-cell sequencing and computational modelling (PIC-seq), showing specific gene signatures in interacting myeloid and CD4^+^ T cells in the TME^22,23^.

As described below in detail, in defined co-cultures, we noted that human antigen-specific CD8^+^ T cells outcompeted non-specific T cells in forming heterotypic clusters with matched antigen-expressing tumour cells. This result, together with the observations described above, prompted us to investigate whether tumour-specific CD8^+^ T cells could be isolated from clinical cancer specimens as heterotypic clusters, and whether they show a distinct biological phenotype and anti-tumour activity.

## Antigen-specific T cell competitiveness

**Fig. 1:**
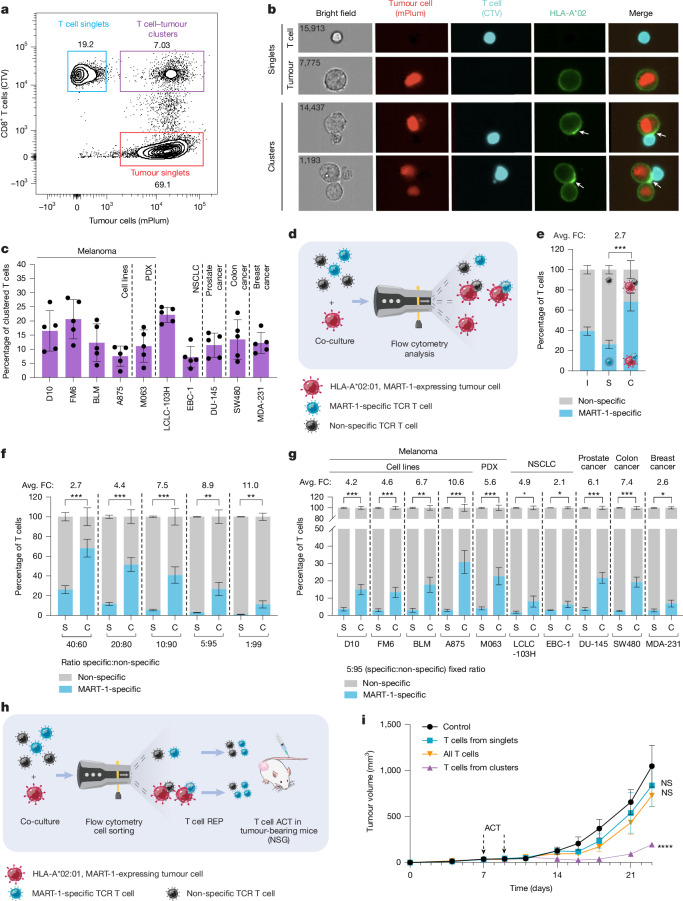
Antigen-specific T cell competitiveness. **a**, FM6 melanoma cells co-cultured for 4 h with T cells were analysed by flow cytometry. **b**, D10 melanoma cells co-cultured for 4 h with T cells were stained and visualized using imaging flow cytometry. The white arrows indicate immunological synapse marker relocalization. Numbers indicate cell identifiers. **c**, The percentage of clustered T cells after 4 h co-culture with different cancer cell lines using 40% MART-1-transduced T cells. *n* = 5 biological replicates (different T cell donors). Data are mean ± s.d. NSCLC, non-small-cell lung cancer. **d**, Diagram of the competition assay: tumour cells were co-cultured with a mixture of MART-1-specific and non-specific T cells and subsequently analysed using flow cytometry. **e**, A875 melanoma cells were co-cultured for 4 h with a 40:60 mix of MART-1-specific:non-specific T cells (input, I). The percentage of MART-1-specific and non-specific T cells in clusters (C) and singlets (S) was assessed using flow cytometry. The average fold change (avg. FC) in MART-1-specific T cells in clusters over singlets was calculated. *P* values were calculated using paired *t*-tests. *n* = 5 biological replicates. Data are mean ± s.d. **f**, A875 melanoma cells were co-cultured for 4 h with different mixtures of MART-1-specific:non-specific T cells; the experiment was performed and analysed as described in **e**. **g**, Co-culture of different cancer cell lines with a 5:95 mixture of MART-1-specific:non-specific T cells; the experiment was performed and analysed as described in **e**. **h**, Diagram of the in vivo experiment: BLM melanoma cells were co-cultured for 4 h with a 20:80 mixture of MART-1-specific:non-specific T cells, and subsequently sorted using FACS. T cells from singlets or clusters were expanded using a rapid expansion protocol (REP). A total of 1.0 × 10^7^ T cells was intravenously injected at day 7 and day 9 into BLM melanoma-bearing mice (NSG), and tumour growth was evaluated. **i**, Tumour growth after ACT with T cells from singlets, T cells from clusters, all T cells or PBS (control). *P* values were calculated using two-way analysis of variance (ANOVA) followed by Tukey’s multiple-comparison test. Significance is indicated compared with the control. *n* = 4 mice per group. Data are mean ± s.e.m. NS, not significant; *P < 0.05, **P < 0.01, ***P < 0.001, ****P < 0.0001. Source Data

**Extended Data Fig. 1:**
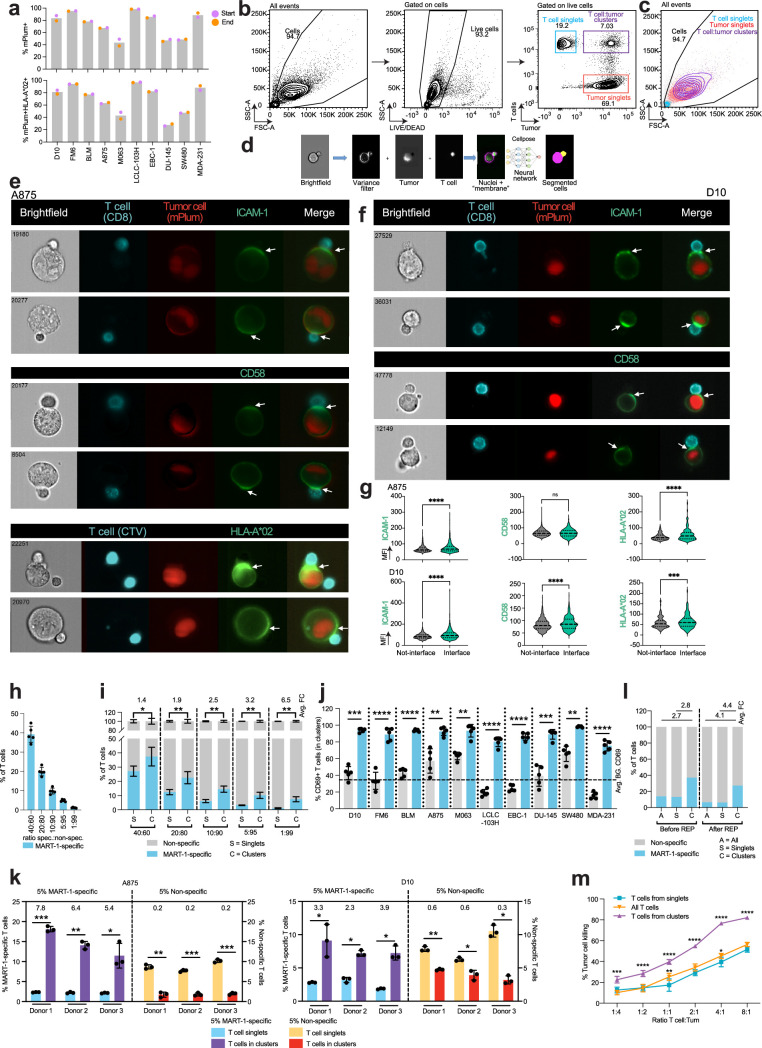
Antigen-specific T cell competitiveness. **a**, Percentage mPlum^+^ and mPlum^+^HLA-A*02:01^+^ cells in the cancer cell line panel, at start and end of experiments in Fig. 1, measured by flow cytometry. **b**, Gating strategy for identifying clusters after 4 h co-culture of tumour cells (mPlum^+^) and CD8^+^ T cells (CTV-stained). Cells were gated based on FSC-A and SSC-A, after which live cells were identified as nearIR-negative. Within live cells, clusters were identified based on mPlum/CTV double-positive cells (also visualized in Fig. 1a). **c**, FSC-A, SSC-A plot showing location of T cell singlets, tumour singlets and T cell:tumour clusters as identified in **(b)**. **d**, Schematic representation of image analysis. ImageStream images were analysed by FIJI. Cellpose was used for segmentation based on nuclear and membranous signals. The membranous signal was obtained by applying a variance filter on the brightfield image. The nuclear signal was obtained by combining the normalized signal from the nuclear markers. From the segmented regions membrane label maps were created, with the interface between cells identified as pixels where different labels touch. Mean intensity of interface versus no interface was measured in the fluorescence channel of interest. **e**, 4 h co-culture of A875 melanoma cells (mPlum^+^) with CD8^+^ T cells (CD8 or CTV-stained) visualized by imaging flow cytometry. White arrows indicate relocalization of indicated markers to the immunological synapse. **f**, 4 h co-culture of D10 melanoma cells with CD8^+^ T cells as in **(e)**. White arrows indicate relocalization of the indicated markers to the immunological synapse. **g**, Quantification of ICAM-1, CD58 and HLA-A*02 mean fluorescence intensity at the T cell:tumour cell interface versus rest of the membrane (not-interface). Representative T cell donor and two melanoma cell lines (D10 and A875) shown. Each data point indicates a tumour cell interacting with a CD8^+^ T cell. Paired *t*-test was used. *n* = 3 biological replicates (different T cell donors, see Supplementary Table 1). **h**, Measured (*y*-axis) compared to expected (*x*-axis) percentage MART-1-specific T cells in input mixes. *n* = 5 biological replicates. Mean ± S.D. **i**, 4 h co-culture of D10 melanoma cells with different mixtures of MART-1-specific:non-specific T cells. Percentage MART-1-specific T cells in clusters (C) versus singlets (S) and Avg. FC were determined. Paired *t*-test was used. *n* = 5 biological replicates. Mean ± S.D. **j**, Quantification of CD69^+^ percentage on MART-1-specific (blue) or non-specific (grey) T cells in clusters. Average CD69^+^ background shown as dotted line. Paired *t*-test was used. *n* = 5 biological replicates. Mean ± S.D. **k**, 4 h co-culture of A875 (left) or D10 (right) melanoma cells with 5:95 or 95:5 mixture MART-1-specific:non-specific T cells. Percentage MART-1-specific or non-specific T cells in clusters versus singlets and Avg. FC were determined. Points represent technical replicates. Paired *t*-test was used. *n* = 3 biological replicates. Mean ± S.D. **l**, Percentage MART-1-specific and non-specific T cells in all T cells (A), singlets (S) and clusters (C) before/after REP. These different populations were used for ACT in BLM melanoma-bearing NSG mice of Fig. 1i. Avg. FC comparing clusters versus singlets or all T cells. **m**, In vitro killing of BLM melanoma cells by the different T cell populations used for ACT. Tumour killing was normalized to untreated tumour cells. Two-way ANOVA, followed by Dunnett’s multiple comparisons test. Significantly increased killing compared to T cells from singlets shown. Mean ± S.D. Mean of three technical replicates. *P < 0.05; **P < 0.01; ***P < 0.001; ****P < 0.0001. Source Data

To study functional interactions between human T cells and tumour cells, we used a matched co-culture model that we established previously^24,25^. We engineered melanoma cells to express both HLA-A*02:01 and the MART-1 tumour antigen, as well as an mPlum fluorescent marker (Extended Data Fig. 1a). CD8^+^ T cells were isolated from healthy donors, retrovirally transduced with a MART-1-specific TCR and labelled with CellTrace Violet (CTV). On the basis of flow cytometry analysis performed after co-culture for 4 h, we observed single tumour cells and single T cells. However, we also noticed a cell population that was positive for both the tumour and T cell labels, suggestive of the formation of heterotypic clusters (Fig. 1a and Extended Data Fig. 1b,c). Using imaging flow cytometry (ImageStream Mark II) we visualized these cell clusters and their immunological synapses (as judged by the significant relocalization of HLA-A*02, ICAM1 and CD58 specifically to the T cell–tumour cell interface; Fig. 1b, Extended Data Fig. 1d–g and Supplementary Table 1). This observation was not limited to melanoma, but was also made for four other cancer indications (Fig. 1c).

These results led us to investigate whether non-specific and antigen-specific T cells differentially engage with tumour cells to form cell–cell conjugates. We admixed non-specific (approximately 60%) and MART-1-specific (approximately 40%) T cells to compete for association with tumour cells. After co-culture for 4 h, we evaluated the contribution of each T cell group to the clusters (Fig. 1d). We observed a 2.7-fold enrichment of MART-1-specific T cells in heterotypic tumour cell clusters compared with singlets (Fig. 1e). We next challenged the system to mimic a more physiological setting such as the TME, in which tumour-reactive T cells are probably under-represented^15,18,26,27^. In all titrations, MART-1-specific T cells outcompeted their non-specific counterparts for cluster formation. Even when specific T cells accounted for only 1% of all T cells, they were up to 11-fold enriched in clusters with tumour cells (Fig. 1f and Extended Data Fig. 1h–i). This competitive advantage of antigen-specific T cells was not limited to melanoma, but reproduced across different cancer indications (Fig. 1g). In all cases, conjugation with tumour cells led to increased activation of antigen-specific T cells, as judged by CD69 induction, compared with non-specific T cells (Extended Data Fig. 1j). To determine the specificity of the system, we also inverted these titrations: when 95% of MART-1-specific T cells were mixed with 5% of non-specific T cells, the latter were depleted from (rather than enriched in) tumour cell clusters (Extended Data Fig. 1k).

To begin exploring preclinical translation of these findings, we determined the relative tumour-controlling potential of single and clustered T cells. After a rapid-expansion protocol (REP), we performed two rounds of adoptive cell transfer (ACT) with the different T cell populations in human-melanoma-bearing immunodeficient NOD-scid *Il2rg*-null (NSG) mice (Fig. 1h). Whereas T cells expanded from singlets showed no tumour control, T cells derived from heterotypic clusters with tumour cells significantly suppressed tumour growth (Fig. 1i and Extended Data Fig. 1l,m). Together, these results indicate that, in defined co-cultures, matched T cells and tumour cells form heterotypic clusters that can form immunological synapses, in which antigen-recognizing T cells outcompete non-specific T cells. When transplanted into mice, T cells from clusters show enhanced tumour control.

## Clinical heterotypic CD8^+^ T cell clusters

**Fig. 2:**
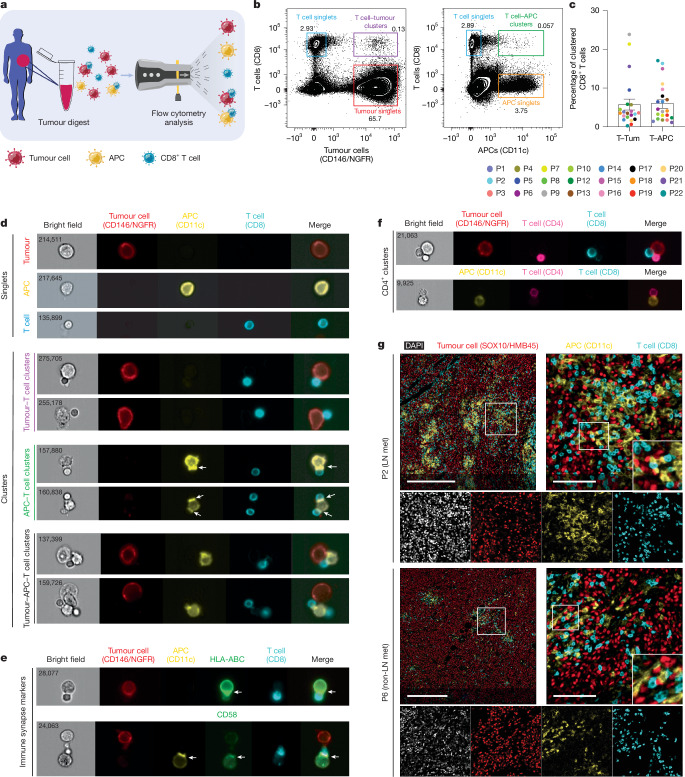
Clinical heterotypic CD8^+^ T cell clusters. **a**, Diagram of tumour sample collection and processing: fresh tumour samples were obtained, cut into small pieces, briefly enzymatically digested, stained and analysed using flow cytometry to identify tumour cells, T cells and APCs. **b**, Representative flow cytometry plots of a melanoma tumour digest (patient 1, P1) processed as in **a**. The tumour digest was stained for the tumour cell markers CD146 and NGFR, the T cell marker CD8 and APC marker CD11c. Plots were obtained from live cells gated as shown in Extended Data Fig. 2a. **c**, The percentage of T cell–tumour cell (T–Tum) and T cell–APC (T–APC) clusters within the total CD8^+^ T cell population. Each coloured point represents an individual patient (*n* = 21). Data are mean ± s.e.m. **d**, The tumour digest (patient 1) from **b** was visualized using imaging flow cytometry. Representative single cells (top) and heterotypic clusters with different compositions (bottom) are shown. The white arrows indicate relocalization of markers to the immunological synapse in T cell–APC clusters. **e**, Tumour digest (patient 6, P6) visualized using imaging flow cytometry. Representative clusters are shown. The white arrows indicate relocalization of immunological synapse markers. **f**, Tumour digest (patient 17, P17) visualized using imaging flow cytometry. Representative heterotypic clusters containing CD4^+^ T cells are shown. In **d**–**f**, numbers indicate cell identifiers. **g**, Multiplex immunofluorescence staining of tissue sections of patient 2 (lymph node metastasis (LN met)) and patient 6 (non-lymph node metastasis). The sections were stained for the tumour cell markers SOX10 and HMB45, T cell marker CD8 and APC marker CD11c. DAPI was included as a nuclear marker. In the top rows, merged images are shown (DAPI is not included for clarity). The white boxes indicate magnified areas. In the bottom rows, channels are separated and correspond to the second images on the top row. *n* = 11 patients; representative patients are shown. Scale bars, 500 μm (**g**, left) and 100 μm (**g**, right). Source Data

**Extended Data Fig. 2:**
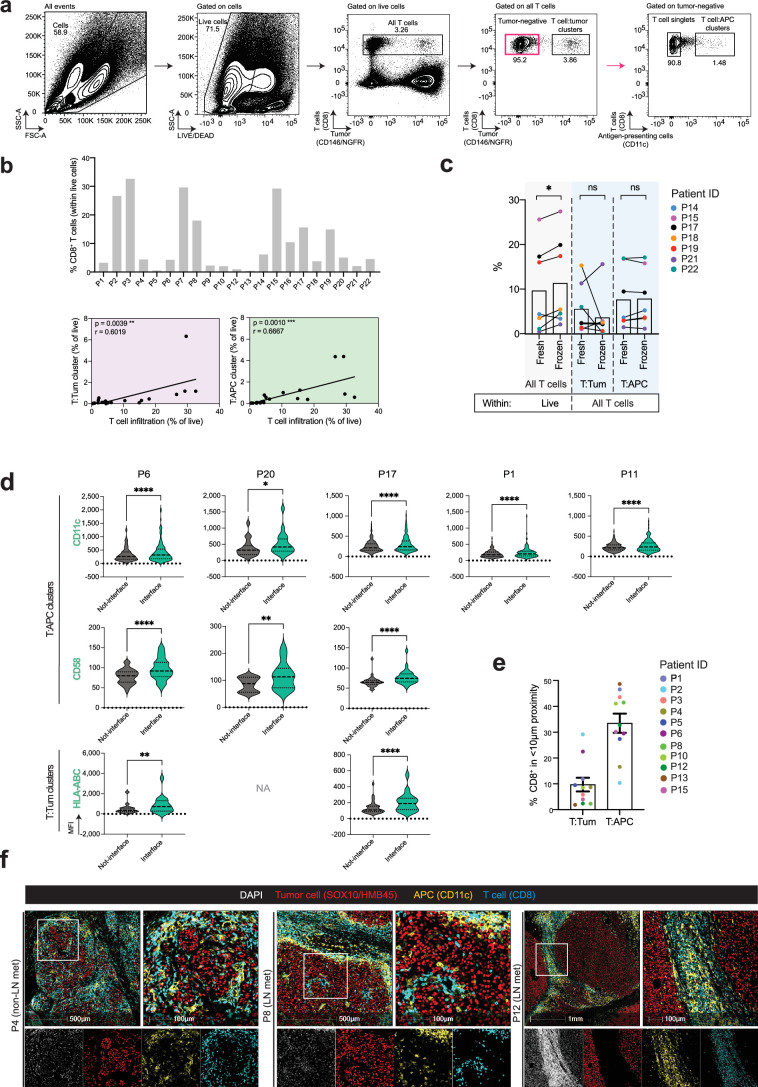
Clinical heterotypic CD8^+^ T cell clusters. **a**, Gating strategy for identification of clusters in clinical melanoma specimens. Cells were gated based on FSC-A and SSC-A, after which live cells were identified as nearIR-negative. From all T cells, first T cell:tumour cell clusters were gated. Second, from the tumour-negative population T cell:APC clusters were identified. Plots in Fig. 2b are both derived from the live cells gate to also visualize tumour and APC singlets. **b**, Percentage CD8^+^ T cell infiltration within live cells (*n* = 21 patients, top) and correlation between percentage infiltration and percentage T:tumour cell (bottom left) or T:APC (bottom right) clusters within live cells. Pearson correlation coefficients. Bars (top) and points (bottom) represent individual patients. **c**, Percentage CD8^+^ T cell infiltration within live cells and percentage T:tumour cell or T:APC clusters within total CD8^+^ T cells comparing fresh and frozen tumour digests. Paired *t*-test was used. Points represent individual patients (*n* = 7), bar represents mean. **d**, Quantification of CD11c, HLA-ABC and CD58 mean fluorescence intensity at the T:APC (CD11c, CD58) or T:tumour cell (HLA-ABC) interface versus rest of the membrane (not-interface). Paired *t*-test was used. Each data point indicates a T:tumour cell or T:APC cluster. *n* = 2-5 patients (see Supplementary Table 1 for all markers and cluster types analysed). **e**, Percentage of CD8^+^ T cells within 10 μM proximity of a tumour cell (T:Tum) or APC (T:APC) in multiplex analyses (*n* = 11 patients). Each coloured point represents an individual patient. Mean ± S.E.M. **f**, Multiplex immunofluorescence stainings on tissue sections of patient 4 (non-lymph node metastasis), patient 8 (lymph node metastasis) and patient 12 (lymph node metastasis). Sections were stained for tumour cell markers SOX10 and HMB45, T cell marker CD8 and APC marker CD11c. DAPI was included as a nuclear marker. In the top row, merged images are shown, DAPI is not included for clarity reasons. White boxes indicate zoom-in areas. In the bottom row, channels are separated and correspond to the second pictures on the top row. *n* = 11 patients, representative patients are shown. *P < 0.05; **P < 0.01; ***P < 0.001; ****P < 0.0001. Source Data

These observations, together with the reported correlations between CD8^+^ T cell–tumour cell proximity and immunotherapy response described above, prompted us to investigate whether heterotypic clusters between CD8^+^ T cells and tumour cells can also be isolated directly from clinical cancer specimens. We analysed a cohort of 21 melanoma metastases from various anatomical sites, including lymph nodes (Supplementary Table 2). After surgical removal, the tissue was cut into small fragments and enzymatically digested for a maximum of 30 min, after which the samples were analysed by flow cytometry using antibodies specific for melanoma (CD146 and NGFR) and T cells (CD8). Owing to the prevalence of APCs in lymph nodes, we also included an APC marker (CD11c). As expected, we identified single cells for each of these populations: CD8^+^ T cells, melanoma cells and APCs. Importantly, we also observed heterotypic CD8^+^ T cell–melanoma cell clusters and CD8^+^ T cell–APC clusters from all patient samples (Fig. 2a–c and Extended Data Fig. 2a). The percentage of heterotypic CD8^+^ T cell clusters within live cells significantly correlated with the degree of T cell infiltration into tumours and was not affected by a freeze–thaw cycle (Extended Data Fig. 2b,c).

ImageStream imaging flow cytometry confirmed these heterotypic cell clusters, comprising one or more CD8^+^ T cells conjugated to either one or more tumour cells and/or one or more APCs (Fig. 2d). The immune synapse markers CD11c, HLA-ABC and CD58 were significantly relocalized to the cell–cell interface (Fig. 2d,e, Extended Data Fig. 2d and Supplementary Table 1). We also identified clusters comprising CD4^+^ T cells, tumour cells and APCs (Fig. 2f). The presence of CD8^+^ clusters was corroborated by multiplex immunofluorescence analysis of the same clinical samples (Fig. 2g and Extended Data Fig. 2e,f). We noted common niches comprising CD8^+^ T cells, tumour cells and APCs in <10 μm vicinity. Together, these results confirm that heterotypic CD8^+^ and CD4^+^ T cell clusters can be detected in, and isolated from, clinical cancer specimens.

## Tumour-reactive CD8^+^ T cells from clusters

**Fig. 3:**
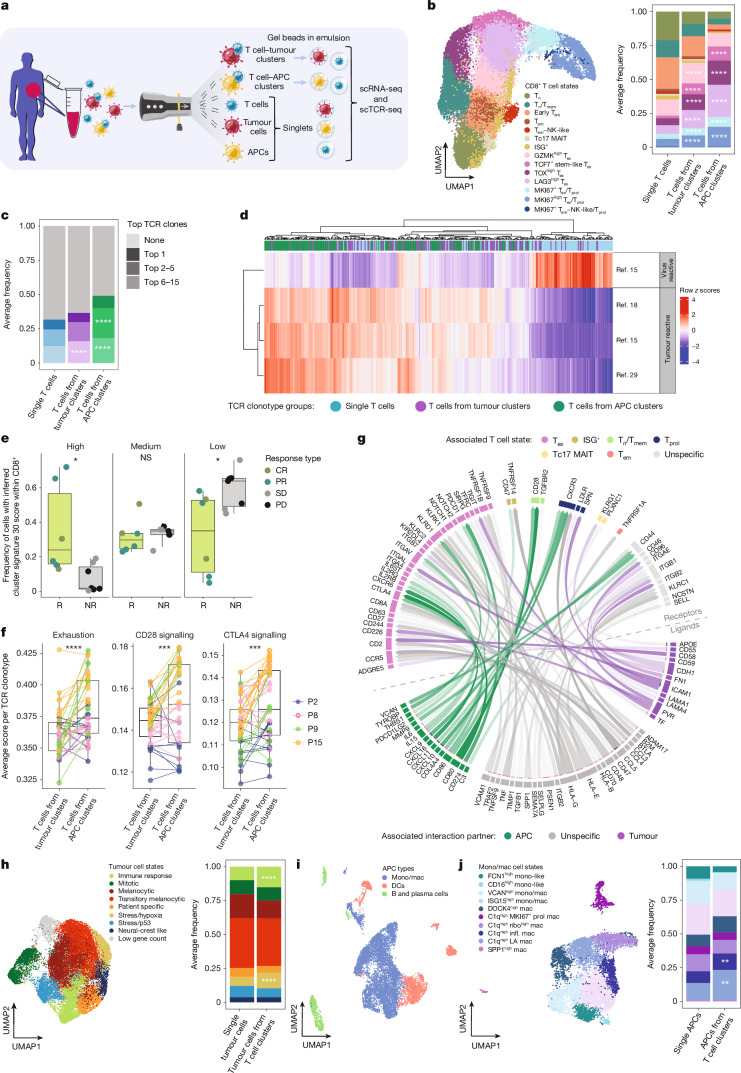
Tumour-reactive CD8^+^ T cells from clusters. **a**, Diagram of the workflow of the scRNA-seq and scTCR-seq analysis. **b**, scRNA-seq UMAP of CD8^+^ T cells, highlighting the main cell states (left) and the average frequencies (right). *n* = 5 patients. Each patient was weighted equally. Tc17, IL-17-producing T cells; MAIT, mucosa-associated invariant; ISG^+^, interferon-stimulated-gene positive cells. Bonferroni-adjusted *P* values were calculated using generalized linear mixed-effects models; significantly enriched cell states in clustered versus single T cells are indicated. **c**, The average frequencies of the top 15 TCR clonotypes in single or clustered T cells analysed as in **b**. **d**, The average tumour- and virus-reactivity gene signature scores per TCR clonotype (≥10 cells) from single or clustered T cells; rows are *z* scored. **e**, CD8^+^ T cell frequencies in a cohort of patients with melanoma treated with TILs^30,31^ by cluster 30 signature-derived tertiles (high, medium and low) in responder (R) and non-responder (NR) baseline tumours (*n* = 13 patients; *n* = 6 (R),* n* = 7 (NR)). CR, complete response; PR, partial response; SD, stable disease; PD, progressive disease. *P* values were calculated using unpaired *t*-tests. **f**, The average signature scores for exhaustion, CD28 and CTLA4 signalling in TCR-matched clonotypes from clustered T cells. The top 10 clonotypes per patient are shown (≥10 cells per cluster group); *n* = 37 matched clonotypes from 4 patients. Each patient (P) is represented by a different colour. *P* values were calculated using paired Wilcoxon signed-rank tests. In **e** and **f**, all datapoints are shown. **g**, The top 50 inferred ligands and their receptor interactions. The arrow transparency reflects inferred ligand signalling activity. ‘Unspecific’ indicates shared ligands or receptors. **h**, scRNA-seq UMAP from melanoma tumour cells, highlighting the main cell states (left) and average frequencies (right). *n* = 5 patients. Analysed as in **b**. **i**, scRNA-seq UMAP of all APC types. *n* = 5 patients. Mono/mac, monocytes and macrophages; DCs, dendritic cells. **j**, scRNA-seq UMAP from monocytes and macrophages, highlighting the main cell states (left) and the average frequencies (right). *n* = 5 patients. Analysed as in **b**. NS, not significant; *P < 0.05, **P < 0.01, ***P < 0.001, ****P < 0.0001. Source Data

**Extended Data Fig. 3:**
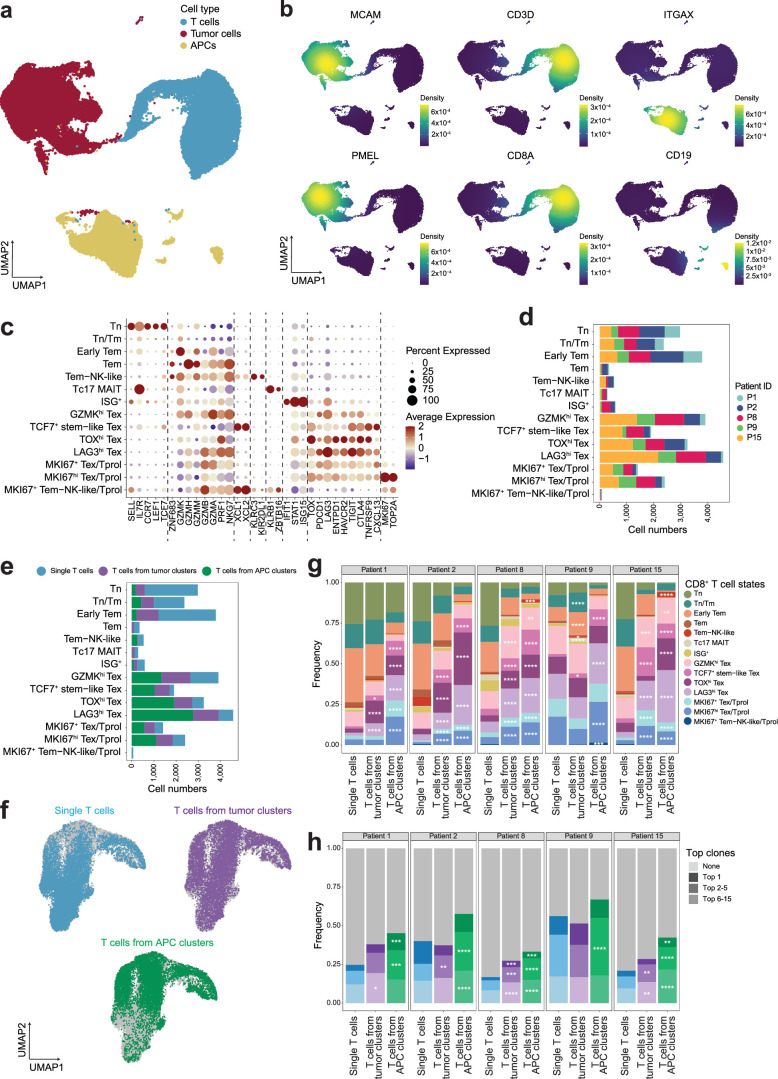
CD8^+^ T cell states and TCR clonality in melanoma clinical samples. **a**, scRNA-seq UMAP of all sequenced cells from melanoma lymph node metastases (*n* = 5 patients), coloured by cell type. **b**, Expression of representative tumour cell, T cell and APC markers projected on UMAP of **(a)**. Colour scale indicates gene-weighted density. **c**, Dotplot showing gene expression of a panel of T cell-related genes indicating different CD8^+^ T cell states. Rows labelled by annotated cell states of Fig. 3b. Colours, average expression of gene; dot size, percentage of cells expressing the gene. **d**, Absolute number of CD8^+^ T cells per annotated cell state subdivided by patient. **e**, Absolute number of CD8^+^ T cells per annotated cell state stratified by origin: single T cells, T cells from tumour clusters or T cells from APC clusters. **f**, Distribution of single T cells and T cells from tumour or APC clusters on the UMAP of sequenced T cells in Fig. 3b. **g**, Frequency of CD8^+^ T cell states in single and clustered T cells per patient (*n* = 5). FDR-adjusted Fisher’s exact test was used. Significantly enriched cell states in clustered versus single T cells indicated. **h**, Frequency of top 15 TCR clonotypes in single or clustered T cells per patient (*n* = 5) analysed as in **(g)**. *P < 0.05; **P < 0.01; ***P < 0.001; ****P < 0.0001. Source Data

Next, we used combined single-cell RNA-sequencing (scRNA-seq) and single-cell TCR sequencing (scTCR-seq) for in-depth comparisons between singlets and clusters for CD8^+^ T cells, tumour cells and APCs. Melanoma specimens were again digested briefly, but this time were separated by fluorescence-activated cell sorting (FACS) to obtain CD8^+^ T cell, tumour cell and APC singlets and clusters (Fig. 3a). The sorting caused most clusters to dissociate into single cells, which were captured into gel beads in emulsion droplets and subjected to sequencing. Combined analysis of all patient specimens (*n* = 5) confirmed three distinct cell types in the clusters as expected: T cells, tumour cells and APCs, consistent with our flow and imaging analyses (Extended Data Fig. 3a,b).

We analysed the CD8^+^ T cell population derived from both singlets and clusters in detail to determine their cell states and TCR clonality, corresponding to two key characteristics of T cell activity. CD8^+^ T cells were annotated according to their respective cell states based on RNA expression profiles of T-cell-related genes and cross-labelling with external single-cell datasets of human CD8^+^ TILs^14,15,20,28^. We identified 14 cell states, showing similarities to cell states described previously^14,15,20,28^ (Fig. 3b and Extended Data Fig. 3c–f), including naive (T_n_, expressing SELL and IL-7R), (early) effector memory (T_em_, expressing GZMH and GZMK), exhausted (T_ex_, expressing PDCD1, TOX, CXCL13, LAG3) and proliferating (T_prol_, expressing MKI67) CD8^+^ T cells. Notably, two subpopulations of proliferating T cells that we identified also expressed exhaustion markers and were therefore termed T_ex_/T_prol_ cells.

We next compared the cell states of T cells derived from singlets and clusters. T cell singlets were enriched for naive and (early) effector memory T cells (T_n_, T_n_/T_mem_, T_em_), whereas T cells derived from both tumour and APC clusters were enriched for exhausted and proliferative cell states (T_ex_, T_ex_/T_prol_) (Fig. 3b, Extended Data Fig. 3g and Supplementary Table 3). We also determined the expansion of the top 15 most-frequent TCR clonotypes^17,26^ (defined as one or more cells with a unique paired α- and β-TCR sequence). This revealed that, relative to single T cells, T cells from both tumour cell and APC clusters were enriched for clonal TCRs (Fig. 3c, Extended Data Fig. 3h and Supplementary Table 3). These results raised the possibility that T cells from clusters are expanded and enriched for tumour-reactive clonotypes.

**Extended Data Fig. 4:**
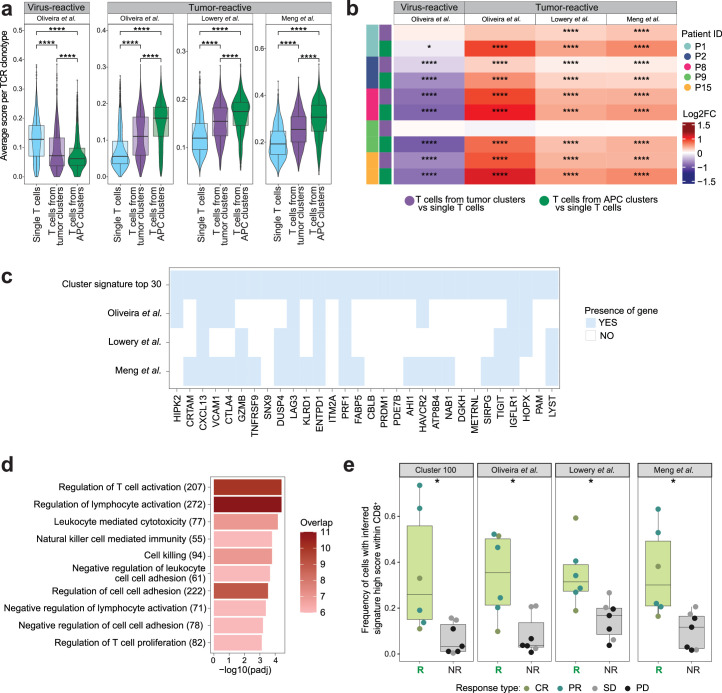
Tumour-reactive CD8^+^ T cells from clusters. **a**, Violin plots of average tumour- and virus-reactivity gene signature scores per TCR clonotype (*n* = 7102 clonotypes from 5 patients) for single or clustered T cells. Used gene signatures can be found in Supplementary Table 4^15,18,29^. Unpaired Wilcoxon signed-rank test was used. Data points beyond the boxplot whiskers are shown as dots. **b**, Heatmap showing log2 fold-change (FC) in average tumour- and virus-reactivity gene signature scores per TCR clonotype for clustered versus single T cells per patient. Unpaired Wilcoxon signed-rank test was used (*n* = 5 patients). **c**, Overlap of the genes from the cluster top 30 signature with the three external tumour-reactivity gene signatures from **(a)**. **d**, Over-representation analysis using the genes from the cluster top 30 signature. Top 10 identified pathways according to significance are visualized. FDR-adjusted one-sided hypergeometric test was used. **e**, Frequency of CD8^+^ T cells with high (top-tertile) signature scores in baseline tumours of responders (R) and non-responders (NR) from an external melanoma TIL-treated patient cohort^30,31^ (*n* = 13 patients; R = 6, NR = 7). Cluster top 100 and the three tumour-reactivity gene signatures from **(a)** were used. CR, complete response; PR, partial response; SD, stable disease; PD, progressive disease. Unpaired *t*-test was used. All data points are shown. *P < 0.05; **P < 0.01; ***P < 0.001; ****P < 0.0001. Source Data

We also assessed the expression of several gene signatures specific for T cell reactivity against tumour cells^15,18,29^. T cell clones originating from clusters showed increased expression of tumour-reactivity signatures (Fig. 3d, Extended Data Fig. 4a,b and Supplementary Table 4). By contrast, single T cell clones exhibited higher expression of a virus reactivity signature, characteristic of bystander T cells^15^. Furthermore, we generated a gene signature derived from clustered T cells that showed both unique and shared features with other tumour-reactivity T cell signatures (Extended Data Fig. 4c and Supplementary Table 4), as well as enrichment for T cell activation, cytotoxicity and cell–cell adhesion gene sets (Extended Data Fig. 4d and Supplementary Table 5). We then projected these cluster T cell (30- and 100-gene) signatures onto an external dataset of melanoma TIL therapy responders and non-responders^30,31^. We observed that the frequency of CD8^+^ TILs with a high cluster signature score at baseline was significantly predictive of therapy response (Fig. 3e and Extended Data Fig. 4e), corroborating the association between T cells from clusters and tumour reactivity. Together, these data indicate that CD8^+^ T cells from clusters, compared with single T cells, show a more exhausted and tumour-reactive phenotype and increased TCR clonality, while they have a distinct RNA profile that predicts TIL therapy response.

## Differential tumour and APC conjugation

We next determined whether CD8^+^ T cells show differential exhaustion profiles after conjugation with APCs compared with tumour cells. To avoid confounding effects, we performed this analysis taking advantage of our scTCR-seq data. Gene signature analysis revealed that T cells with identical TCRs from top-expanded clonotypes were more exhausted when conjugated to APCs than when conjugated to tumour cells^32^ (Fig. 3f and Supplementary Tables 4 and 5). Moreover, while several ligand–receptor pairs were shared between CD8^+^ T cell–tumour cell and T cell–APC conjugates (including HLA-CD8), the latter clusters showed more co-modulatory interactions, such as between CD80/CD86 and CTLA-4/CD28 and between PD-L1/2 and PD-1 (Fig. 3f,g). For T cell–tumour cell interactions, we observed several adhesion interactions, including between CD58 and CD2 and between ICAM1 and ITGAL (Fig. 3g). These results reveal that TCR-matched T cells show more exhaustion and co-modulation when conjugated to APCs than to tumour cells. Notably, exhausted T cells in the TME usually contain the largest fraction of tumour-reactive T cells that can be reinvigorated after treatment^15,18,29,30^.

## Distinct APCs and tumour cells in clusters

**Extended Data Fig. 5:**
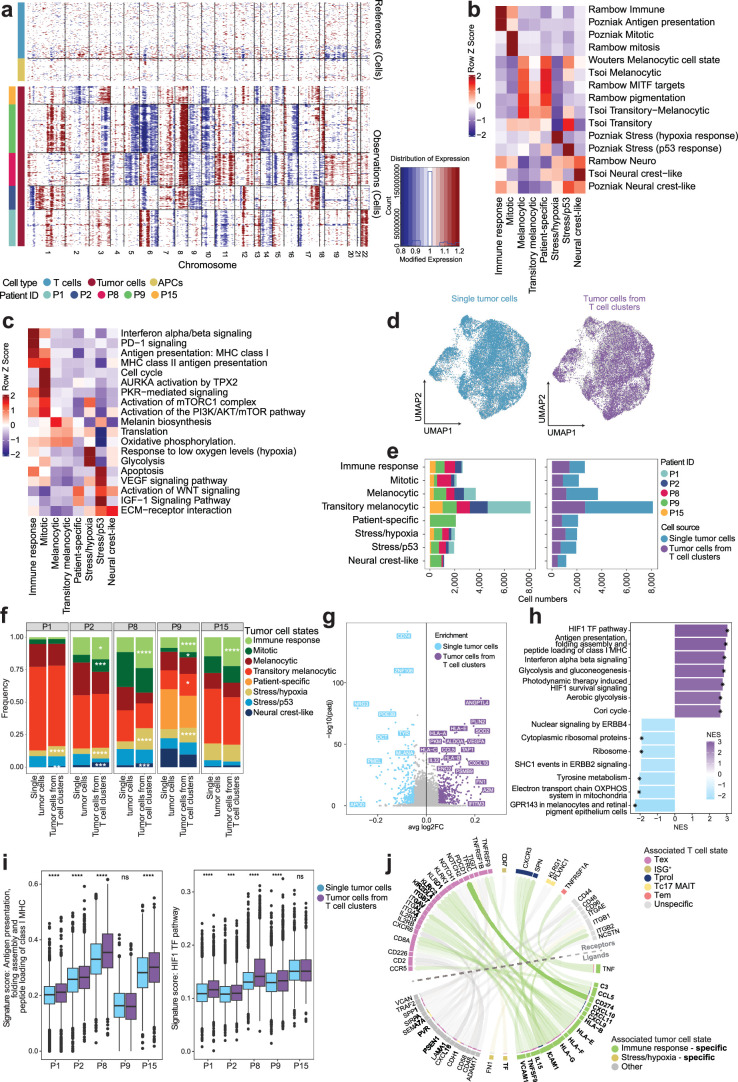
Tumour cell states in melanoma clinical samples. **a**, InferCNV-estimated copy number variations (CNVs) in tumour cells and as a reference T cells and APCs, shown per patient. Gains and losses of specific regions of the DNA are indicated in red and blue, respectively. **b**, Heatmap of AUCell scores for several external melanoma gene signatures^4,33–35^ across annotated tumour cell states. Columns labelled by annotated cell states of Fig. 3h. Rows are z-scored. **c**, Heatmap showing AUCell scores of enriched pathways across annotated tumour cell states. Visualized as in **(b)**. Used pathways in Supplementary Table 5. **d**, Distribution of single tumour cells and tumour cells from T cell clusters on the UMAP of sequenced tumour cells in Fig. 3h. **e**, Absolute number of tumour cells per annotated cell state subdivided by patient (left) or stratified by origin (right): single tumour cells or tumour cells from T cell clusters. **f**, Frequency of cell states in single tumour cells or tumour cells from T cell clusters per patient (*n* = 5). FDR-adjusted Fisher’s exact test was used. Significantly increased cell state frequencies of tumour cells from T cell clusters versus single tumour cells are indicated. **g**, Volcano plot comparing tumour cells from T cell clusters with single tumour cells. Blue, genes enriched in single tumour cells; purple, genes enriched in tumour cells from T cell clusters. Representative selection of genes labelled. Bonferroni-adjusted MAST test was used, with patient of origin as latent variable (*n* = 5 patients). **h**, Gene set enrichment analysis using differentially expressed genes in tumour cells from clusters and single tumour cells. Top seven most upregulated and downregulated pathways shown. Significant pathways (FDR-adjusted *P*-value < 0.05) are indicated with an asterisk. Used gene sets in Supplementary Table 5. **i**, For the two most enriched pathways in tumour cells from T cell clusters in **(h)**, the gene signature score of each cell was plotted per patient (*n* = 23,772 cells from 5 patients). Unpaired Wilcoxon signed-rank test was used. Data points beyond the boxplot whiskers are shown as dots. **j**, Circos plot of top 30 inferred ligands and their receptor interactions focusing on differential signalling of different tumour cell states towards T cells. Arrow transparency reflects predicted ligand signalling activity. Ligands or receptors were assigned to specific tumour or T cell states respectively based on expression level differences. Ligands for tumour cell states enriched in T cell clusters are highlighted. If the ligand is coloured and bold, it is uniquely associated with that cluster enriched group. Ligands labelled as “other” are not associated with the cluster enriched groups. Receptors shared between all or multiple T cell states are labelled unspecific. *P < 0.05; **P < 0.01; ***P < 0.001; ****P < 0.0001. Source Data

After this characterization of clustered T cells, we next investigated whether there is any preferential conjugation of CD8^+^ T cells to specific tumour cell and APC subpopulations. We annotated tumour cell and APC subtypes on the basis of existing gene signatures and marker genes^4,31,33–42^. In agreement with previous studies on melanoma heterogeneity^4,33,35^, we observed a broad spectrum of melanoma cell states, including melanocytic and neural-crest like (Fig. 3h, Extended Data Fig. 5a–e and Supplementary Table 5). When analysing their representation in clusters, we observed that specific melanoma subpopulations were enriched, particularly those associated with immune response (for example, antigen presentation and interferon signalling) and stress/hypoxia response (for example, HIF signalling) (Fig. 3h, Extended Data Fig. 5f–i and Supplementary Table 3). Cell–cell communication analysis revealed that specifically the T-cell-interacting, immune-response-associated melanoma subpopulation showed higher expression of ligands mediating T cell attraction (for example, CCL5–CCR5, CXCL9/10–CXCR3), immune synapse formation (for example, HLA-CD8 and ICAM1–ITGAL) and immune modulation (for example, PD-L1–PD-1) (Extended Data Fig. 5j).

**Extended Data Fig. 6:**
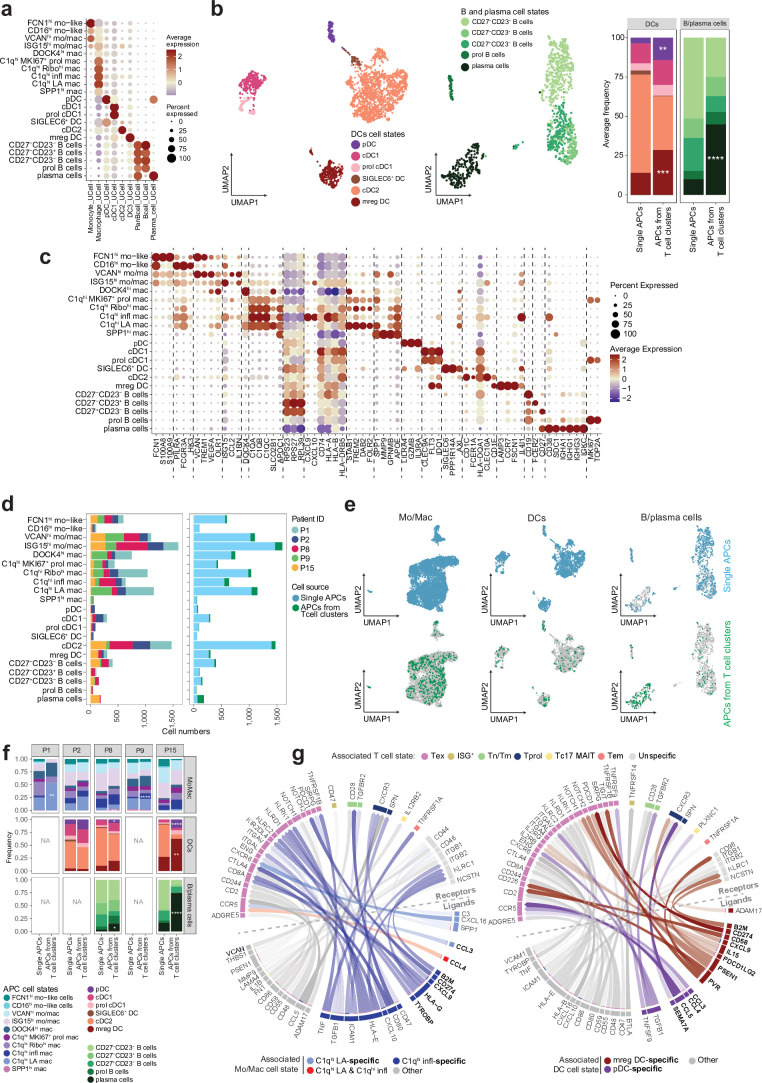
APC cell types and states in melanoma clinical samples. **a**, Dotplot of UCell scores for a set of defined APC types to discriminate APC types in Fig. 3i. Rows labelled by annotated cell states. Colours, average UCell score; dot size, percentage of cells expressing the UCell geneset. **b**, scRNA-seq UMAP of sequenced DCs and B/plasma cells highlighting the main cell states (left) and average frequencies (right) (*n* = 5 patients). Each patient was weighted equally (*n* = 3 for DCs and *n* = 2 for B/plasma cells). DC and B/plasma cell states were annotated based on marker genes and signatures^31,36–38,40–42^. Bonferroni-adjusted *P* values from generalized linear mixed-effects models; significant increase in cell state frequency of APCs in T cell clusters versus single APCs is indicated. Patients were excluded if <20 cells were detected in the respective APC type from T cell clusters. **c**, Dotplot showing gene expression of a panel of APC-related genes discriminating different APC cell states. Rows labelled by annotated cell states of Fig. 3j and (**b**). Colours, average expression of gene; dot size percentage of cells expressing the gene. **d**, Absolute number of APCs per annotated cell state subdivided by patient (left) or stratified by origin (right): single APCs or APCs from T cell clusters. **e**, Distribution of single APCs and APCs from T cell clusters on the UMAP of sequenced monocytes and macrophages, DCs and B/plasma cells as provided in Fig. 3j or (**b**) respectively. **f**, Frequency of cell states in single APCs or APCs from T cell clusters per patient (*n* = 5). FDR-adjusted Fisher’s exact test was used. Significantly increased cell state frequencies of APCs in T cell clusters versus single APCs are indicated. Patients were excluded if <20 cells were detected in the respective APC type from T cell clusters. **g**, Circos plots of top 30 inferred ligands and their receptor interactions focusing on the differential signalling of different APC states towards T cells. Mono/macrophages and DCs were analysed separately. Arrow transparency reflects predicted ligand signalling activity. Ligands or receptors were assigned to specific APC or T cell states respectively based on expression level differences. Ligands for APC states enriched in T cell clusters are highlighted. If the ligand is coloured and bold, it is uniquely associated with that cluster enriched group. Ligands labelled as “other” are not associated with the cluster-enriched groups. Receptors shared between multiple or all T cell states are labelled unspecific. *P < 0.05; **P < 0.01; ***P < 0.001; ****P < 0.0001. Source Data

We performed a similar enrichment analysis for APCs, based on the identification of monocytes/macrophages, dendritic cells (DCs) and B/plasma cells isolated from the same clinical samples (Fig. 3i), all of which we annotated on the basis of previous studies^31,36–42^ (Fig. 3j and Extended Data Fig. 6a–e). The monocytes/macrophages comprised a range of phenotypes, including CD16^high^ monocyte-like cells and C1q^high^ macrophages (Fig. 3j). We observed that, among all states, specifically the C1q^high^ lipid-associated and C1q^high^ inflammatory macrophages were enriched in CD8^+^ T cell clusters (Fig. 3j, Extended Data Fig. 6f and Supplementary Table 3). These subpopulations showed higher expression of ligands mediating T cell attraction (for example, CCL4–CCR5, CXCL9/10–CXCR3) and co-modulation (for example, PD-L1–PD-1 and CD80–CTLA-4/CD28) (Extended Data Fig. 6g). Likewise, we found enrichment of subpopulations of DCs (particularly plasmacytoid DCs and mature DCs enriched in regulatory molecules, also known as mregDCs) and B cells (particularly plasma cells) in T cell clusters. These enriched DC groups were associated with similar predicted ligand–receptor interactions (such as CCL4–CCR5, CXCL9–CXCR3 and PD-L1–PD-1) (Extended Data Fig. 6g). Collectively, these results show that CD8^+^ T cells preferentially bind to specific subpopulations of both APCs and tumour cells, communicating through specific ligand–receptor pairs.

## Enhanced killing by T cells from clusters

**Fig. 4:**
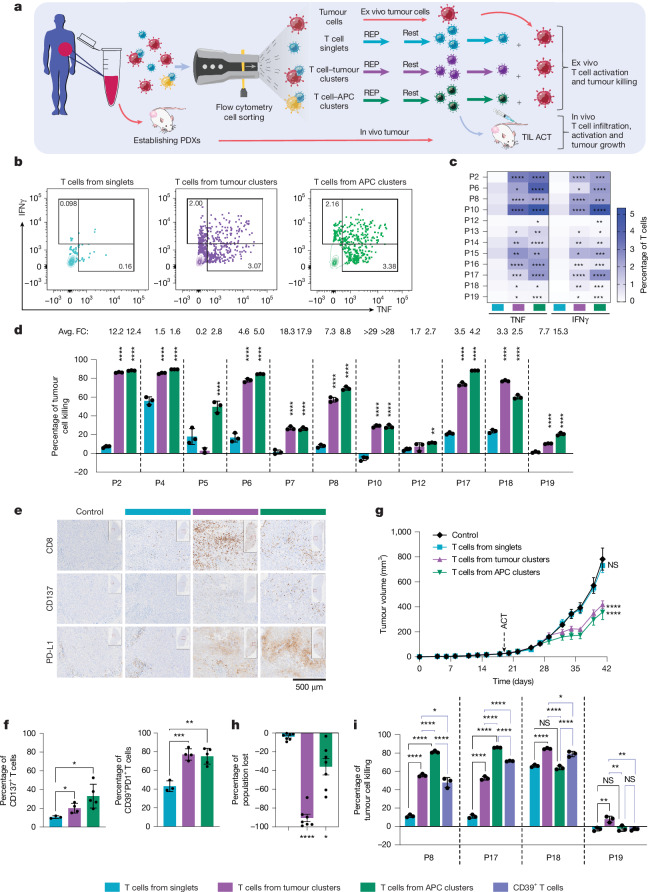
Enhanced killing by T cells from clusters. **a**, Schematic of the ex vivo and in vivo experiments: sorted singlet and clustered T cells were expanded using REP, rested and co-cultured with autologous tumour cells or injected into matched PDX-bearing mice. **b**, Representative flow cytometry plots showing cytokine production by T cells from singlets or clusters after 4 h co-culture with tumour cells (patient 2, P2). **c**, Cytokine production by T cells from singlets or clusters after 4 h co-culture with tumour cells. *n* = 12 patients. The mean of at least two technical replicates is shown. *P* values were calculated using unpaired *t*-tests versus singlets. **d**, Tumour killing by T cells from singlets or clusters (*n* = 11 patients), normalized to untreated tumour cells. The points represent technical replicates.* P* values were calculated using two-way ANOVA followed by Dunnett’s multiple-comparison test. Significantly increased killing and average fold change compared with singlets is shown. Data are mean ± s.d. **e**, Representative immunohistochemistry for CD8, CD137 and PD-L1 in PDX tumours from hIL-2-NOG mice (patient 8, P8), 2 weeks after ACT. *n* = 5 mice per group. Scale bar, 500 μm. **f**, Flow cytometry analysis of T cell activation in PDXs from **e**, measured as the percentage of CD137^+^ and CD39^+^PD1^+^ cells. *P* values were calculated using unpaired *t*-tests. *n* = 5 mice per group; 3 mice were not included owing to insufficient material. Data are mean ± s.d. **g**, PDX tumour growth (patient 8) in NSG mice receiving ACT with T cells from singlets, clusters or PBS (control). * P* values were calculated using two-way ANOVA followed by a Tukey’s multiple-comparison test. Significance was calculated versus the control. *n* = 10 mice per group, except for T cells from tumour clusters, for which *n* = 9. Data are mean ± s.e.m. **h**, The percentage of singlet and clustered T cells lost after single-cell gating (*n* = 7 patients). The points represent patients. Statistical analysis was performed using paired *t*-tests versus singlets. Data are mean ± s.e.m. **i**, Tumour killing by T cells from singlets, clusters or CD39^+^ cells (*n* = 4 patients) normalized as in **d**. * P* values were calculated using one-way ANOVA, followed by Tukey’s multiple-comparison test. The black lines show singlet versus clustered T cells; and the blue lines show clustered versus CD39^+^ T cells. Data are mean ± s.d. In **c**, **d** and **i**, each patient is indicated with P and a number. NS, not significant; *P < 0.05, **P < 0.01, ***P < 0.001, ****P < 0.0001. Source Data

**Extended Data Fig. 7:**
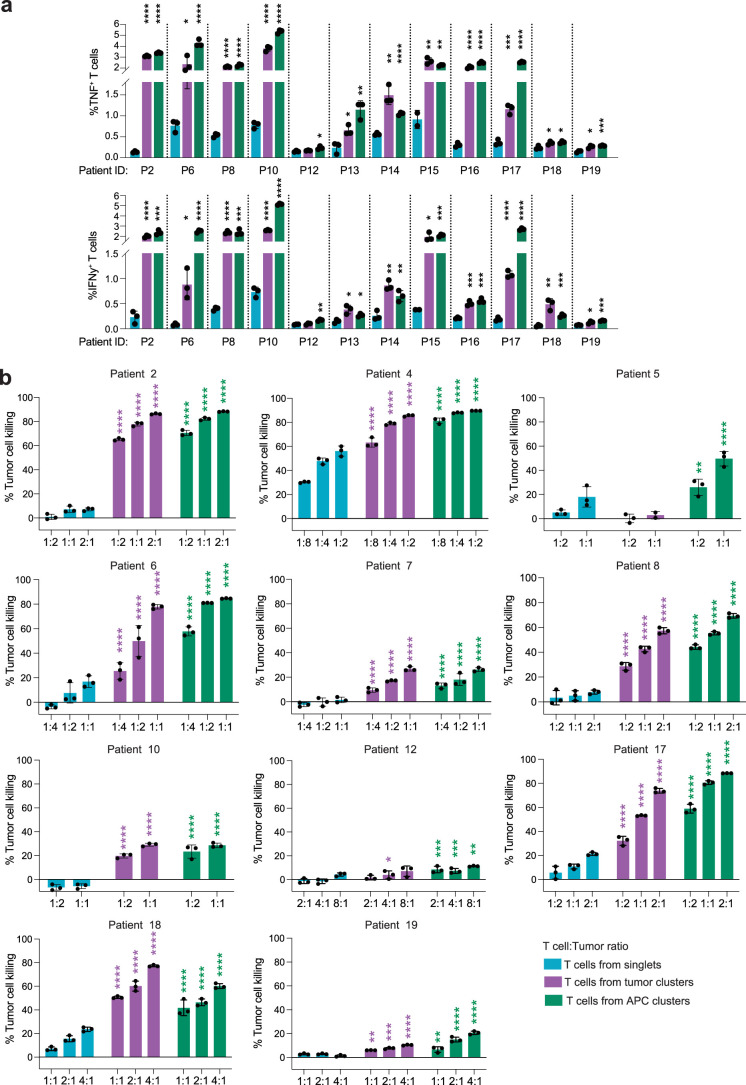
Enhanced killing by T cells from clusters. **a**, Quantification of cytokine production by expanded CD8^+^ T cells derived from singlets (blue), tumour cell clusters (purple) or APC clusters (green) after a 4 h co-culture with autologous tumour cells (*n* = 12 patients). Points represent technical replicates. Unpaired *t*-test versus T cells from singlets was used. Mean ± S.D. **b**, Tumour killing by expanded CD8^+^ T cells derived from either singlets, tumour cell clusters or APC clusters (*n* = 11 patients), normalized to untreated tumour cells. Points represent technical replicates. On the *x*-axis, the T cell:tumour cell ratios are shown. Two-way ANOVA, followed by Dunnett’s multiple comparisons test was used. Significantly increased killing compared to T cells from singlets from the same ratio is indicated. Mean ± S.D. *P < 0.05; **P < 0.01; ***P < 0.001; ****P < 0.0001. Source Data

The results above show that CD8^+^ T cell clusters can be isolated from clinical samples and that they harbour several features predicting enhanced tumour reactivity, which we put to the test. We again isolated T cell singlets and clusters from melanoma digests using FACS but, this time, the sorting step was followed by a REP. After a resting period, the T cells were used to treat autologous melanoma cells ex vivo (Fig. 4a and Supplementary Tables 6 and 7). After 4 h exposure to tumour cells, production of the cytokines IFNγ and TNF was significantly increased in T cells from clusters compared with in T cells from singlets, indicative of higher activation (Fig. 4b,c and Extended Data Fig. 7a). We next determined the tumour-killing potential of T cells from singlets and clusters. Autologous melanoma cells were established from the clinical samples and co-cultured with T cells for several days. As a measure of cell killing, we performed CellTiter-Blue assays, and untreated tumour cells were used as controls. The killing capacity of CD8^+^ T cells derived from tumour cell clusters was increased in 9 out of 11 patients compared with T cell singlets; for T cells from APC clusters, this was seen in 11 out of 11 patients. Relative to T cell singlets, T cells from tumour cell clusters showed over eightfold higher killing activity and T cells from APC clusters were more than ninefold more active (Fig. 4d and Extended Data Fig. 7b).

**Extended Data Fig. 8:**
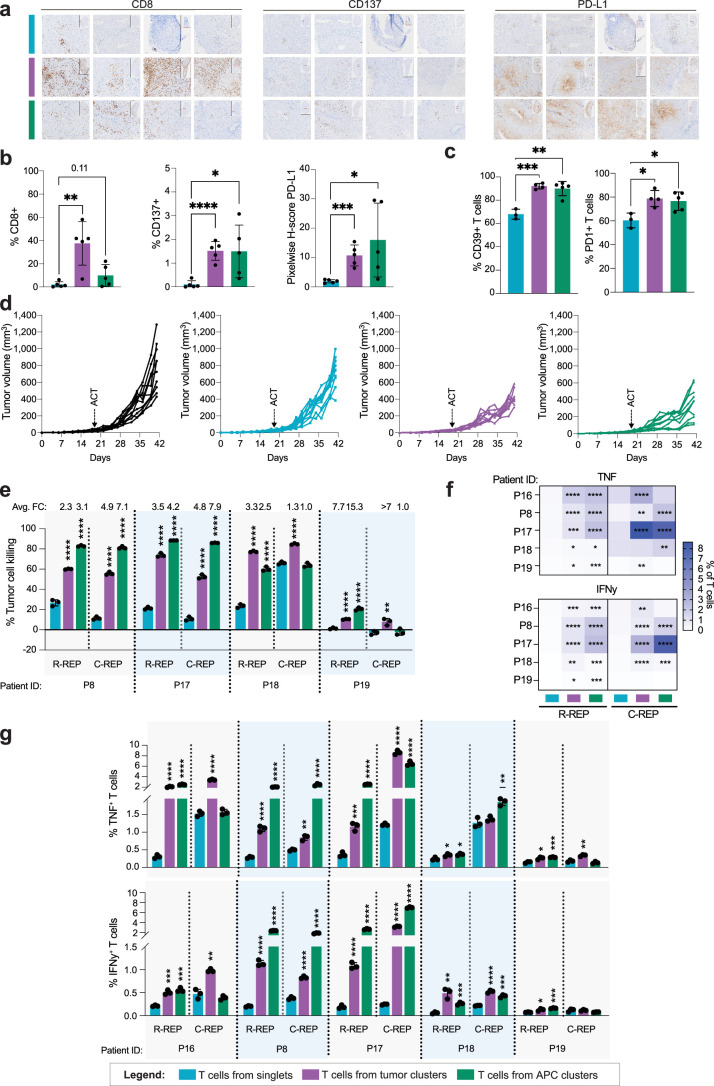
CD8^+^ T cells from clusters retain their tumour-reactive capacity in vivo and after a clinical rapid expansion protocol. **a**, Representative IHC for CD8, CD137 and PD-L1 in PDX tumours from hIL2-NOG mice (patient 8), 2 weeks post-ACT with T cells from singlets, tumour cell clusters or APC clusters. One additional mouse in Fig. 4e. *n* = 5 mice/group. **b**, Quantification of IHC shown in Fig. 4e and (**a**). The whole slide was quantified using Qupath. CD8/CD137: percentage of positive cells within all detected cells. PD-L1: pixelwise H-score (0, no expression; 300, maximum expression). Unpaired *t*-test was used. *n* = 5 mice/group. Mean ± S.D. **c**, Flow cytometry analysis of T cell activation in PDXs from Fig. 4e and (**a**), measured as percentage CD39^+^ or PD1^+^ positive cells, for T cells from singlets, tumour cell clusters and APC clusters. Unpaired *t*-test was used. n = 5 mice/group; 3 mice not included due to insufficient material for flow cytometry (two from singlet, one from tumour cell cluster group). Mean ± S.D. **d**, Spider plots of PDX tumour growth in ACT-treated NSG mice receiving T cells from singlets (blue), T cells from tumour clusters (purple), T cells from APC clusters (green) or PBS (control, black). Each line is an individual mouse. *n* = 10 mice/group; except T cells from the tumour clusters *n* = 9. **e**, Tumour killing by CD8^+^ T cells expanded with research-REP (R-REP) or clinical REP (C-REP) derived from either singlets, tumour cell clusters or APC clusters (*n* = 4 patients). Tumour killing was normalized to untreated tumour cells. Points represent technical replicates. Unpaired *t*-test was used. Significantly increased killing and average fold-change (Avg. FC) compared to T cells from singlets is indicated. Mean ± S.D. **f**, Heatmaps showing cytokine production by R-REP or C-REP expanded CD8^+^ T cells derived from either singlets, tumour cell clusters or APC clusters after 4 h co-culture with autologous tumour cells (*n* = 5 patients). Mean of 3 technical replicates shown. Unpaired *t*-test versus T cells from singlets. Significant increases in cytokine production compared to T cells from singlets is indicated. **g**, Bar plots of technical replicates from **(f)**, showing cytokine production. Mean ± S.D. *P < 0.05; **P < 0.01; ***P < 0.001; ****P < 0.0001. Source Data

We next set out to examine the therapeutic potential of these clustered T cells in two independent mouse experiments and in a clinical TIL REP protocol. First, we performed ACT with patient TILs that were expanded using a REP. Expanded TILs were subsequently inoculated into hIL-2-NOG mice^43^ (for optimal in vivo T cell support) carrying a matched patient-derived melanoma xenograft (PDX). ACT was performed with either T cell singlets or T cells from tumour or APC clusters, which were characterized for in vivo T cell infiltration and activation. We observed that T cells from tumour cell clusters showed increased infiltration, while a similar trend was observed for T cells from APC clusters (Fig. 4e and Extended Data Fig. 8a,b). Moreover, corroborating our ex vivo data, we found significantly increased activation of T cells from clusters relative to T cell singlets, as judged by upregulation of CD137, PD-1, CD39 and increased PD-L1 expression by tumour cells (indicative of IFNγ secretion by active neighbouring T cells) (Fig. 4e,f and Extended Data Fig. 8a–c). As the T cell toxicity observed in this model due to the high IL-2 levels precluded analysis of tumour growth, we also set up an independent mouse experiment using PDX-bearing NSG mice. Whereas adoptively transferred T cell singlets had no effect, T cells from both tumour and APC clusters significantly delayed tumour growth (Fig. 4g and Extended Data Fig. 8d). Thus, T cells from clusters are strongly enriched for tumour killing activity also in vivo.

**Extended Data Fig. 9:**
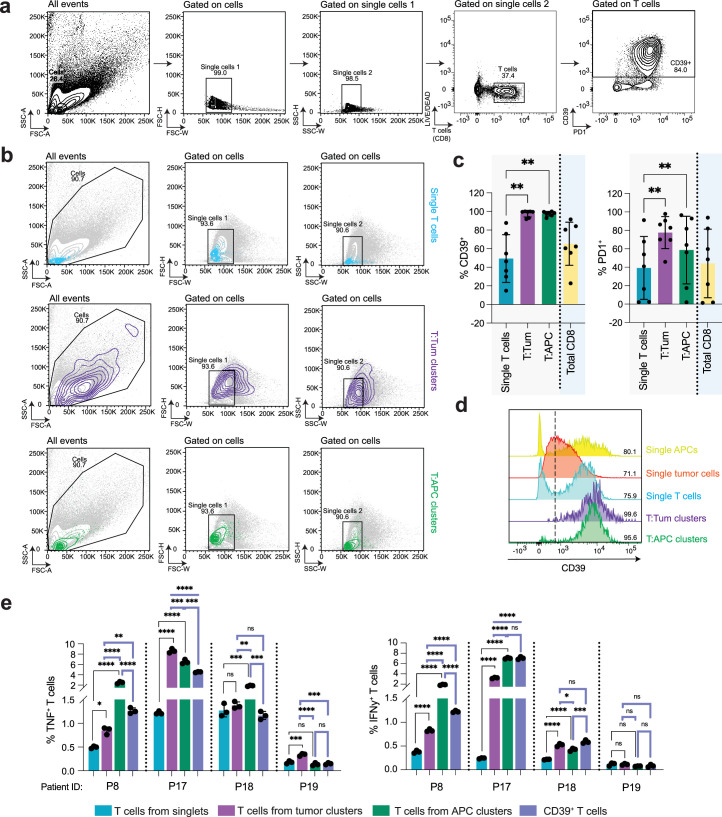
Heterotypic CD8^+^ T cell clusters are a distinct population compared to single CD8^+^CD39^+^ T cells. **a**, Gating strategy for identification of single CD8^+^CD39^+^ T cells in clinical melanoma specimens. Cells were gated based on FSC-A and SSC-A, after which single cells were identified based on FSC-W against FSC-H and SSC-W against SSC-H. From single cells, nearIR-negative live CD8^+^ T cells were identified in which CD39^+^ T cells were gated. PD1 was included as an additional marker in this staining panel. **b**, Projection of single T cells (blue), T:Tum clusters (purple) and T:APC clusters (green) on the first three gating plots of **(a)**. Cluster populations identified in the same sample using the gating strategy shown in Extended Data Fig. 2a. The cells gate in the first plot was increased in size to accommodate clusters. **c**, Frequency of CD39^+^ (left) or PD-1^+^ (right) cells in single T cells, T:Tum clusters and T:APC clusters. Total CD8^+^ T cells, identified using the gating strategy in **(a)**, included as a reference. Points represent patients (*n* = 7). Paired *t*-test was used. Mean ± S.D. **d**, Representative flow cytometry histogram of CD39 expression in single APCs, single tumour cells, single T cells, T:Tum clusters and T:APC clusters. Dotted line separates negative (left) and positive (right) cells. Values indicate percentage CD39^+^ cells. **e**, Quantification of cytokine production by expanded CD8^+^ T cells derived from either singlets, tumour cell clusters, APC clusters or CD39^+^ cells after a 4 h co-culture with autologous melanoma cells (*n* = 4 patients). One-way ANOVA, followed by Tukey’s multiple comparisons test. Points represent technical replicates. Black lines: singlet versus clustered T cells; blue lines: clustered versus CD39^+^ T cells. Mean ± S.D. *P < 0.05; **P < 0.01; ***P < 0.001; ****P < 0.0001. Source Data

Second, we adjusted our REP to make it more compatible with the current clinical TIL REP^44^. We found that T cells derived from clusters retained their functionality and power to significantly outperform singlets in tumour-killing activity (Extended Data Fig. 8e–g). We also benchmarked our cluster-enriched TIL product to current methods using cell-surface markers to enrich for tumour-reactive single T cells, particularly PD-1 and CD39^16,17,26,27,45–49^. However, the use of single-cell gates caused an almost complete loss of T cell–tumour cell clusters (average 91% loss) and a profound decrease in T cell–APC clusters (average 36% loss) (Fig. 4h and Extended Data Fig. 9a,b), highlighting that these clusters represent a unique cell population. Most cell clusters were positive for both CD39 and PD-1 (Extended Data Fig. 9c). Notably, CD39 is not a unique T cell marker, as it is expressed also by tumour cells and/or APCs (Extended Data Fig. 9d). Whereas sorting for CD8^+^CD39^+^ T cells also enriched for tumour-reactive T cells, in 4 out of 4 patients, at least one of the cluster-derived T cell groups outperformed them in tumour cell killing (Fig. 4i and Extended Data Fig. 9e).

**Extended Data Fig. 10:**
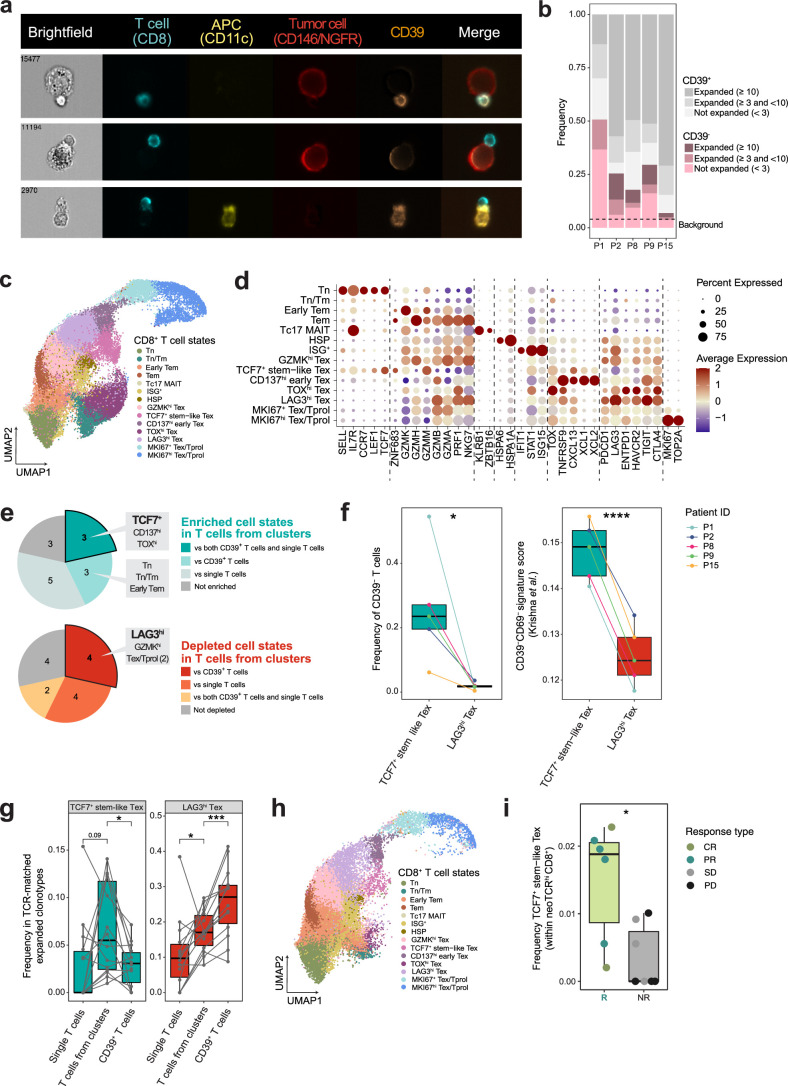
Heterotypic CD8^+^ T cell clusters contain CD39^−^ T cells and are enriched with favourable TCF7^+^ stem-like exhausted T cells compared to single CD8^+^CD39^+^ T cells. **a**, Imaging flow cytometry of tumour digest (patient 18) showing: (i) a CD39^**+**^ T cell in a cluster with a CD39^−^ tumour cell (top row); (ii) CD39^−^ T cells in a cluster with a CD39^+^ tumour cell or APC (middle and bottom rows, respectively). **b**, Frequency of CD39^+^ (grey) and CD39^**−**^ (pink) T cells in clusters across patients used for single cell analyses. CD39 status was determined from scRNA-seq data, using each cell’s *ENTPD1* expression and the average of its ten closest neighbours. Expanded ( ≥ 3 cells/clonotype) and non-expanded (<3 cells) CD39^+^ and CD39^**−**^ T cells were visualized. Expanded subdivided into two groups: ≥3 and <10 or ≥ 10 cells. Dotted line: average background of predicted CD39^**−**^ T cells in CD8^+^CD39^+^-sorted samples. **c**, scRNA-seq UMAP of CD8^+^ T cells from Fig. 3b (*n* = 5 patients), supplemented with CD8^+^CD39^+^-sorted single T cells from two of those patients (P8 and P15). Main CD8^+^ T cell states are highlighted, including two new states: CD137^hi^ early Tex and HSP (Heat Shock Proteins). **d**, Dotplot showing gene expression of a panel of T cell-related genes indicating different CD8^+^ T cell states. Rows labelled by annotated cell states provided in **(c)**. Colours, average expression of gene; dot size, percentage of cells expressing the gene. **e**, Pie charts visualizing enriched and depleted cell states in CD8^+^ T cells from clusters compared to sorted single CD8^+^CD39^+^ T cells or single CD8^+^ T cells (*n* = 2 patients). Cell states enriched/depleted in both comparisons are separately grouped. Each pie chart contains all 14 cell states defined in **(c)**. Bonferroni-adjusted *P* values from generalized linear mixed-effects models. **f**, Frequency of CD39^**−**^ T cells (left) or the CD39^**−**^CD69^**−**^ signature score (right) in TCF7^+^ stem-like Tex and LAG3^hi^ Tex CD8^+^ T cells (*n* = 5 patients). This plot does not include sorted CD8^+^CD39^+^ samples. The used gene signature for CD39^**−**^CD69^**−**^ T cells was obtained from Krishna *et al*. ^50^ and can be found in Supplementary Table 4. Paired *t*-test was used. Quantification of CD39 status as in **(b)**. **g**, Frequency of TCF7^+^ stem-like Tex and LAG3^hi^ Tex cell states in TCR-matched expanded clonotypes across single CD8^+^ T cells, CD8^+^ T cells from clusters and sorted single CD8^+^CD39^+^ T cells (*n* = 14 matched clonotypes from 2 patients). Expanded clonotypes contain ≥ 10 cells per respective subpopulation. Each connected line represents a different TCR clonotype. Paired Wilcoxon signed-rank test was used. **h**, UMAP visualization of label-transferred CD8^+^ T cells from Barras *et al*. (GSE221553)^31^. The annotated cell states and embedding visualized in **(c)** were used as a reference. **i**, Frequency of TCF7^+^ stem-like Tex within neoTCR^hi^ CD8^+^ T cells from Barras *et al*. (GSE221553)^31^ in TIL responder (R) and non-responder (NR) baseline tumours (*n* = 13 patients, R = 6, NR = 7). Annotated label-transferred cells from **(h)** were used. Cells within the top-tertile of the neoTCR score were considered neoTCR^hi^. CR, complete response; PR, partial response; SD, stable disease; PD, progressive disease. Unpaired *t*-test was used. For **(f)**, **(g)** and **(i)** all data points are shown as dots. *P < 0.05; **P < 0.01; ***P < 0.001; *P* < 0.0001. Source Data

Although T cells from clusters contained CD39^+^ cells, we noted that they also included a CD39^−^ T cell population (ranging from 7 to 51%) (Extended Data Fig. 10a,b). These CD39^−^ T cells were even observed in expanded clonotypes (ranging from 28 to 76% within CD39^−^ T cells from clusters), suggesting tumour reactivity. Using single-cell analysis, we next compared the cell states between sorted single CD8^+^CD39^+^ T cells and CD8^+^ T cells from clusters. We observed that T cells from clusters were enriched for persistence-associated^50–52^, memory-like and early dysfunctional cell states (including TCF7^+^ stem-like T_ex_ cells, which are more frequently CD39^−^), whereas sorted CD39^+^ T cells were enriched for terminally exhausted cells (LAG3^high^ T_ex_ cells) (Extended Data Fig. 10c–f and Supplementary Tables 3 and 4). This was also true for TCR-matched T cells (Extended Data Fig. 10g). Analysis of an annotated external melanoma dataset^31^ revealed that the cluster-enriched TCF7^+^ stem-like T_ex_ cell state was significantly associated with clinical TIL response (Extended Data Fig. 10h,i).

We conclude from these analyses together that CD8^+^ T cells derived from cell clusters exert significantly greater anti-tumour activity than single T cells both ex vivo and in vivo. Moreover, they retain their potential in an expansion protocol resembling clinical TIL REP and are enriched in a favourable TCF7^+^ stem-like exhausted cell state compared with other enrichment strategies.

## Discussion

To study the TME, much effort has focused on the analysis of single cells by flow cytometry and sequencing, which has greatly advanced our understanding of its composition and complexity^18–20,36^. However, cells can engage in stable homotypic and heterotypic interactions in vivo^21–23,53–55^. This study shows that heterotypic CD8^+^ T cell clusters containing tumour cells and/or APCs can be retrieved directly from clinical cancer specimens from various anatomical sites, including lymph nodes. We demonstrate that, compared with single T cells, these clustered T cells possess biologically distinct features and are strongly enriched for tumour reactivity.

Using flow and imaging analyses, we show that CD8^+^ T cells are conjugated to several types of APCs, including monocytes/macrophages, DCs and B cells. These clusters are stable enough to withstand a freeze–thaw cycle and, although some interactions may result from dissociations and (re)associations ex vivo, our single-cell RNA analysis revealed that the cell–cell associations do not occur in a random manner. Indeed, for all these APC types, we observe enrichment of specific subpopulations in clusters. For example, for monocytes/macrophages, we find enrichment of particularly C1q^high^ lipid-associated and C1q^high^ inflammatory subtypes in CD8^+^ T cell conjugates, which are similar to macrophage subtypes associated with clinical responses to TIL therapy^31^. Likewise, among different melanoma cell states present in the TME, there is significant enrichment of those characterized by high antigen presentation, IFN signalling and stress/hypoxia-response signalling, which could be a cause or consequence of T cell interaction. Furthermore, both APC and tumour subpopulations enriched in clusters exhibit higher expression of ligands that are critical for immune synapse formation, T cell attraction and immune modulation upon conjugation with T cells.

In-depth analysis of clustered CD8^+^ T cells themselves also revealed several unique features. First, compared with single T cells, we observed that clustered CD8^+^ T cells are more exhausted and proliferative, while they also show clonal expansion of their TCRs. Notably, exhausted T cells within the TME typically have the largest proportion of tumour-reactive T cells, which can be reactivated through treatment^15,18,29,30^. Second, whereas single T cells show features consistent with virus specificity typical of bystander cells, clustered T cells are strongly enriched for tumour-reactive signatures. Third, a T cell signature that we derived from clusters is predictive in baseline samples for patient response to TIL therapy^30,31^. Fourth, taking advantage of our scTCR-seq analysis, we observed that T cells carrying identical top clonal TCRs show more exhaustion and co-modulation when conjugated to APCs than when conjugated to tumour cells. All of these results are in agreement with, and extend, previous observations on cellular conjugates, for example, homotypic and heterotypic circulating-tumour-cell clusters with different properties^21^, interacting CD4^+^ T cell–APC clusters characterized by PIC-seq^22,23^ and the importance of spatial positioning of CD4^+^–CD8^+^ T cell–DC triads^56,57^.

The characterization of the biology of tumour–immune cellular conjugates described above revealed that clustered CD8^+^ T cells display many features predictive of tumour-reactivity in other studies^14,15,18,20,29,51,58^. These include competitive engagement with tumour cells, enrichment of exhausted phenotypes, specific tumour-reactive gene expression programs, increased TCR clonality and association with response to TIL therapy. This was corroborated in several different preclinical models. First, CD8^+^ T cells from clusters expanded in a REP show on average ninefold increased ex vivo killing activity compared with single T cells, which was associated with increased production of IFNγ and TNF. Second, ACT with CD8^+^ T cells from clusters into two models of autologous PDX-bearing mice showed significantly more T cell infiltration, T cell activity and tumour control compared to treatment with single T cells. Third, we corroborated these results in a REP resembling the current clinical TIL REP^44^.

Benchmarking these results to single CD8^+^CD39^+^ T cells^16,26,27,46–49,59^, we demonstrate that clusters also contain considerable numbers of CD39^−^ T cells, even in expanded clonotypes, and that they are enriched in favourable memory-like and TCF7^+^ stem-like exhausted cell states. Putting this into context, it was recently shown in TIL products that the presence of memory-progenitor CD39^−^ stem-like cells within neoantigen-specific TILs is associated with clinical response and TIL persistence, in contrast to a terminally differentiated CD39^+^ cell state^50^. This is consistent with previous reports demonstrating that the presence of less-differentiated memory-like, early dysfunctional or stem-like TILs at baseline is associated with improved outcomes after immune checkpoint blockade or TIL therapy, as well as prolonged response duration^3,20,51,52,60^. Together, these results demonstrate that T cells from clusters differ substantially from single CD39^+^ T cells and possess features related to persistence that are relevant for the development of enhanced TIL therapy.

In conclusion, we demonstrate that heterotypic CD8^+^ T cell clusters represent a cell population with distinct biological characteristics and a marked enrichment for tumour reactivity. We propose that these clusters, which are often excluded from cell sorting and therefore neglected in single-cell sequencing procedures, represent a unique cell population allowing for better understanding of functional tumour–immune cell interactions and warranting preclinical exploration. Our findings not only support the potential for improving TIL therapy, but also merit exploring therapeutic strategies based on the isolation of TCRs from clustered T cells.

## Methods

### Cell lines

Human cancer cell lines were obtained from the Peeper lab repository. They were short-tandem-repeat profiled to confirm identity and tested mycoplasma-negative at the start of in vitro experiments. Cell lines were transduced with lentivirus to express HLA-A*02:01-MART1-mPlum plasmid as described previously^25^. D10, FM6, BLM, A875, M063 and MDA-MB-231 (referred to as MDA-231) cell lines were cultured in Dulbecco’s modified Eagle’s medium (DMEM; 41966052, Gibco) with 10% FBS (3101120, Sigma-Aldrich) and 100 U ml^−1^ penicillin–streptomycin (15140122, Invitrogen). LCLC-103H, EBC-1, DU-145 and SW480 cells were cultured in RPMI (21875034, Thermo Fisher Scientific) with 10% FBS and 100 U ml^−1^ penicillin–streptomycin. For REP, the suspension cell line EBV-JY was used, which was cultured in IMDM (CA IMDM-A, Capricorn Scientific) supplemented with 10% FBS and 100 U ml^−1^ penicillin–streptomycin.

### Primary human CD8^+^ T cell isolation, transduction and culture

Primary CD8^+^ T cells used in in vitro experiments with cell lines were isolated from healthy donor blood (from buffy coats). In brief, PBMCs were isolated by density centrifugation using Ficoll (11743219, Thermo Fisher Scientific) (2,500 rpm, 15 min, no break). CD8^+^ T cells were positively isolated with Dynabeads (11333D, Invitrogen) and activated for 48 h in a precoated plate with anti-hCD3 and anti-hCD28 (16-0037-85/16-0289-85, eBioscience), 5 mg per well in 24-well plates at 10^6^ cells per ml. CD8^+^ T cells were then retrovirally transduced in retronectin-coated (T100B, Takara) plates with the MART-1-specific TCR (2,000*g*, 1.5 h, no break). For the first 2 days after activation, primary CD8^+^ T cells were cultured in RPMI with 10% human serum (H3667, Sigma-Aldrich) and 100 U ml^−1^ penicillin–streptomycin, with IL-2, IL-7 and IL-15 (100 IU ml^−1^, 10 ng ml^−1^, 10 ng ml^−1^ respectively) (Proleukin, Novartis; 11340075, Immunotools; 11340155, Immunotools). T cells were then refreshed three times a week with RPMI containing 10% FBS, 100 U ml^−1^ penicillin–streptomycin and 100 IU ml^−1^ IL-2.

### Patient samples

Resected tumour material was collected from patients with melanoma undergoing surgery at the Netherlands Cancer Institute/Antoni van Leeuwenhoek Hospital (NKI-AvL) (Supplementary Table 2). The study was approved by the Medical Ethical Review Board of the NKI-AvL (under studies B16MEL, IRBm23-029) and performed in compliance with the ethical regulations. All of the patients provided prior informed consent to use their anonymized data and tumour material for research, including publication of the results in a manuscript.

### Patient tumour digestion

To obtain tumour digests, freshly obtained patient tumours were cut in small pieces and incubated in prewarmed RPMI medium supplemented with pulmozyme (12.6 µg ml^−1^; Roche), collagenase (1 mg ml^−1^; 17104-019, Thermo Fisher Scientific) and a pan-caspase inhibitor (Q-VD-Oph, 50 μM; or Z-VAD, 5 μg ml^−1^; S7311, Selleckchem; sc-3067, Santa Cruz Biotechnology) at 37 °C in a spinning rotor for a maximum of 30 min. The sample was then passed through a 100-μm filter, washed with RPMI containing 10% FBS and frozen in FBS + 10% DMSO until further processing.

### In vitro T cell–tumour cell line co-cultures

Before the start of the co-culture, primary CD8^+^ T cells were labelled with CTV (C34557, Invitrogen) or carboxyfluorescein succinimidyl ester (CFSE; C34554, Invitrogen) according to the manufacturer’s instructions. Tumour cell lines and pre-labelled CD8^+^ T cells were counted and seeded in a non-tissue-culture-treated 96-well V-bottom plate (781601, Brand) at a 2:1 tumour:T cell ratio for standard flow cytometry and at a 1:1 ratio for image-based flow cytometry assays (50,000 tumour and 25,000 or 50,000 T cells, respectively). Co-culturing was performed in 100 μl per well with 50 μl of tumour cell medium and 50 μl of T cell medium with IL-2. In standard assays, cells were co-cultured for 4 h and subsequently analysed by flow cytometry. For competition assays, non-specific and MART-1-specific T cells were mixed at the indicated ratios before the start of co-culture, based on the measured transduction efficiency. After most co-cultures, the percentage of MART-1-specific T cells in the populations of interest was determined by staining for the mouse TCR β-chain. For the experiment in which the 5:95 and 95:5 ratios (MART-1-specific:non-specific) were studied together (Extended Data Fig. 1k), the T cells were sorted after transduction to obtain a pure MART-1-specific T cell population. Before the co-culture, MART-1-specific T cells were stained with CTV and non-specific T cells with CFSE, after which they were mixed at the ratios described above to perform the co-culture.

### Flow cytometry and cell sorting

For flow cytometry, the culture medium was removed and cells were washed with 0.1% BSA in PBS. For surface staining, cells were stained with the indicated antibodies diluted in 0.1% BSA in PBS for 30 min on ice in the dark. For intracellular staining, cells were stained using the FOXP3 kit (00-5523-00, Invitrogen) according to the manufacturer’s instructions. A list of the antibodies used is provided in Supplementary Table 8. After staining, cells were washed twice with 0.1% BSA in PBS and measured using a BD LSRFortessa, BD LSR-II SORP or BD FACSymphony A5 SORP flow cytometer with the FACSDiva (v.8 or v.9) acquisition software. Data were analysed using Flowjo (v.10.8.1). For primary human tumour samples, previously frozen tumour digests were thawed and washed twice with RPMI, supplemented with 10% FBS and 1:1,000 benzonase nuclease (purity, >90%) (70746-3, VWR). Cells were washed an additional time with 0.1% BSA in PBS after which they were stained with antibody mix for 30 min on ice in the dark. After staining, cells were washed twice with 0.1% BSA in PBS before flow cytometry or sorting. When indicated, the samples were washed and stained with 2% BSA in PBS and sorted in 2% FBS in PBS. Cell sorting was performed using a BD FACSAria Fusion cell sorter with an 85, 100 or 130 µM nozzle depending on the size of cells and clusters sorted. Sorted cells were collected in RPMI supplemented with 20% FBS, before proceeding to downstream processing. To prevent mislabelling of non-interacting cells as clusters, we ran the samples at a low cell concentration and measured at a low event rate. Moreover, the Fusion cell sorter has several quality-control measures to prevent sorting of these events (for example, electronic aborts and precision mode). As described previously^61–63^, cell sorting disrupted physical connections between cells in clusters, which was confirmed by microscopy, with the vast majority of cells being singlets post-sort, allowing further downstream single-cell analyses.

### ImageStream analysis

For ImageStream analysis, samples were processed following the flow cytometry staining procedure described above and diluted to 1.0 × 10^7^ cells per ml in 0.1% BSA in PBS after the final wash. Cells were analysed using ImageStream Mark II system with INSPIRE acquisition software (v.200.1.681.0). Obtained data were processed using IDEAS software (v.6.3 or v.6.4). Data were exported as individual OME .tiff^64^ files and combined into multichannel stack .tiff files using the custom made program ImageStreamCombiner. Image analysis workflows were developed in FIJI (v.2.14)^65^ with the steps performed using CLIJ (v.2.5)^66^ for GPU processing. Cellpose (v.2 or v.3)^67^ was used for cell segmentation as follows. For the in vitro samples, a nuclear and membranous signal served as the input, whereby the membranous signal was obtained by applying a variance filter (radius 2 pixels) on the bright-field image and the nuclear signal was obtained by combining the normalized signals from the T cell and tumour marker channels (Extended Data Fig. 1d). For patient-derived samples, cellpose was performed on a single cytosolic/membranous input channel: a combination of all normalized fluorescence channels and the normalized variance-filtered bright-field channel. After segmentation the resulting labels were contracted with 2 pixels. Cell types (tumour cell, T cell and/or APC) were separated by *k*-means clustering (IJ-Plugins toolkit v.2.3), with the intensities of the fluorescence channels and the cell area as input. The clusters were then classified as cell types by comparing their average marker intensity. Further analysis was focused on 1:1 clusters of two different cell types (larger clusters and non-interacting cells were excluded). The membrane was estimated as the outer 3 pixels of the segmented cells. Touching regions between two different cell types were regarded as interfaces, while the rest of the membrane was considered ‘not an interface’. The intensity of the marker of interest in or outside the interface was measured as the mean of the region. Details on ImageStream experiments are provided in Supplementary Table 1. Scripts for image analysis are available at GitHub (https://github.com/BioImaging-NKI/ImageStreamCombiner and https://github.com/BioImaging-NKI/ImageStreamAnalysis).

### Multiplex staining and analysis

#### Automated multiplex staining on the Discovery Ultra Stainer

Before multiplex staining, 3-µm slides were cut on TOMO slides. The slides were then dried overnight and stored at 4 °C. Before a run was started, the slides were baked for 30 min at 70 °C in an oven. Staining was performed on the Ventana Discovery Ultra automated stainer, using the Opal 6-Plex Detection Kit (50 slides kit, Akoya Biosciences, NEL871001KT). The protocol starts with baking for 28 min at 75 °C, followed by dewaxing with Discovery Wash using the standard setting of 3 cycles of 8 min at 69 °C. Pretreatment was performed using Discovery CC1 buffer for 64 min at 95 °C, after which Discovery Inhibitor was applied for 8 min to block endogenous peroxidase activity. Specific markers were detected consecutively on the same slide using the following antibodies: anti-CD8 (C8/144B, M7103, DAKO, 1:50, 2 h at room temperature), anti-CD4 (SP35, 104R-16, Cell Marque, 1:25, 2 h at room temperature), anti-CD69 (EPR21814, ab233396, Abcam, 1:100, 1 h at room temperature), anti-CD11c (D3V1E, CST45581S, Cell Signaling, 1:50, 1 h at room temperature), anti-SOX10 (BC34, BCARACI3099C, Biocare Medical, 1:20, 2 h at room temperature), anti-HMB45 (PMEL/melanoma gp100, 38815, Cell Signaling, 1:400, 2 h at room temperature) and anti-HLA-A (EP1395Y, ab52922, Abcam, 1:2,000, 2 h at room temperature). Anti-SOX10 and anti-HMB45 were incubated at the same time by making a mixture of the two antibodies. Each staining cycle was composed of four steps: primary antibody incubation, secondary antibody mouse (PI-2000-1, Vector laboratories, 1:100, 32 min at room temperature) or rabbit (31460, Invitrogen, 1:250, 32 min at room temperature), OPAL dye incubation (OPAL480, OPAL520, OPAL570, OPAL620, OPAL690, OPAL780, 1:40 or 1:50 dilution as appropriate for 32 min or 1 h at room temperature) and an antibody denaturation step using CC2 buffer for 20 min at 95 °C. Cycles were repeated for each new antibody to be stained. DAPI (FP1490, Akoya, 1:10, 12 min at room temperature) was stained manually afterwards. After the run was finished, slides were washed with demineralized water and mounted with Fluoromount-G (Southern Biotech, 0100-01) mounting medium.

#### Scanning of multiplexed slides with PhenoImager HT

After staining, the slides were imaged using the PhenoImager HT automated imaging system (Akoya). Scans were made with the MOTIF unmixing protocol, using the InForm software v.3.0. The MOTIF images were unmixed into eight channels: DAPI, OPAL480, OPAL520, OPAL570, OPAL620, OPAL690, OPAL780 and autofluorescence.

#### Image analysis using HALO software

The HALO software (v.4.0.5107.357, Indica Labs) was used for image analysis. Analysis was focused on DAPI, CD8, CD11c and SOX10/HMB45. On the basis of tumour area, regions of interest were selected together with a pathologist using the annotation tool. The Indica Labs HighPlex FL v.4.2.14 analysis algorithm was used for analysis using AI nuclei segmentation. Regions of interest were analysed and both the summary data and cell object data were exported in comma-separated value files using the export manager in HALO. Value files were imported into Python (v.3.12) using Pandas (v.2.2.3). Values included the classification and centroid position. Some cells were triple or double positive and needed to be reclassified for further analysis. SOX10/HMB45^+^CD8^+^ double-positive and SOX10/HMB45^+^CD8^+^CD11c^+^ triple-positive cells were changed to unclassified. SOX10/HMB45^+^CD11c^+^ double-positive cells were reclassified as SOX10/HMB45^+^, as the CD11c is often present on membranes that protrude into SOX10/HMB45-positive tissue and cause false-positive classification for CD11c. CD8^+^CD11c^+^ double-positive cells were reclassified as CD8^+^ for the same reason. Nearest-neighbour analysis was performed using scikit-learn (v.1.5.2). For each cell the distance to the nearest SOX10/HMB45-, CD11c- and CD8-positive cell was determined. CD8^+^ cells were counted based on their vicinity to SOX10/HMB45- and CD11c-positive cells. A cut-off of 10 µm was used to define direct proximity as the size of the cells is approximately 10 µm. For downstream analysis, CD8^+^ T cells within <10 μm of SOX10/HMB45-positive or <10 μm of both SOX10/HMB45- and CD11c-positive cells were defined as T cell–tumour cell clusters, similar to our flow cytometry gating strategy in Extended Data Fig. 2a. CD8^+^ T cells within <10 μm to CD11c^+^ cells were defined as T cell–APC clusters.

### scRNA-seq and scTCR-seq

Tumour digests were thawed, stained and sorted as described above. Five populations were sorted from live cells: tumour singlets (NGFR/CD146^+^); tumour–CD8^+^ T cell clusters (NGFR/CD146^+^CD8^+^); APC–CD8^+^ T cell clusters (NGFR^−^CD146^−^CD11c^+^CD8^+^), CD8^+^ T cell singlets (NGFR^−^CD146^−^CD11c^−^CD8^+^) and APC singlets (NGFR^−^CD146^−^CD11c^+^CD8^−^). For two patients, CD8^+^CD39^+^ T cells were sorted separately from single live cells. Singlets were pooled together during sorting at a ratio of 1:1:1. If the number of clusters was low, they were kept as separate samples. If sufficient numbers of clusters were sorted (>40,000 clusters), they were hashtagged with TotalSeq-C0251 (T cell–tumour clusters, 394661, BioLegend) or with TotalSeq-C0252 (T cell–APC clusters, 394663, BioLegend) and subsequently pooled 1:1. Both CD8^+^CD39^+^ single T cell samples were also hashtagged using the same antibodies and pooled 1:1. For hashtagging, sorted cells were washed once with 2% BSA in PBS and incubated with the hashtagging antibody for 30 min on ice. After hashtagging, cells were washed an additional two times with 0.04% BSA in PBS, after which they were pooled. Cells that did not need hashtagging were washed twice with 0.04% BSA in PBS, before proceeding to single-cell 5′ sequencing library preparation.

The Chromium Controller and Chromium X platform of 10x Genomics were used for single-cell partitioning and barcoding. Each cell’s transcriptome was barcoded during reverse transcription, pooled cDNA was amplified and single-cell 5′ gene expression (GEX), V(D)J and feature barcode (FB) Libraries were prepared according to the manufacturer’s protocols (CG000330 and CG000331, 10x Genomics). All libraries were quantified and normalized based on library QC data generated on the Bioanalyzer system according to the manufacturer’s protocols (G2938-90321 and G2938-90024, Agilent Technologies). On the basis of the expected target cell counts, a balanced library subpool of samples was composed for SC5′ GEX, V(D)J and FB libraries. Library subpools were quantified by quantitative PCR (qPCR), according to the KAPA Library Quantification Kit Illumina Platforms protocol (KR0405, KAPA Biosystems). Based on the qPCR results, a final sequencing pool was composed. Paired-end sequencing was performed on the NovaSeq 6000 Instrument (Illumina) using the NovaSeq 6000 Reagent Kits v1.5 100 cycles (20028401, 20028319, 20028316 Illumina), using 28 cycles for read 1, 10 cycles for read i7, 10 cycles for read i5 and 90 cycles for read 2.

### Processing and analysis of scRNA-seq and scTCR-seq data

#### Processing of single-cell data

Sequence alignment was performed with CellRanger (v.7.0.1) using the human genome GRCh38 as a reference to obtain gene expression and TCR sequence data from the samples. For patients 2 and 8, the CD8^+^ T cell–tumour cell clusters and CD8^+^ T cell–APC clusters were pooled and sequenced with Totalseq-C hashtags as described above and processed together using the Cell Ranger multi-run functionality.

For all samples, the gene expression data from the CellRanger output was loaded using Seurat (v.4.4.0)^68^. The pooled samples from patients 2 and 8 are separated using the antibody capture matrix. We generated density plots of hashtag expression, determined the local minimum and identified hashtag-positive cells. Cells expressing both hashtags were filtered out. Moreover, cells containing <200 gene counts, >8,000 gene counts and a percentage of mitochondrial gene expression >15% were filtered out for quality reasons. A total of 71,867 cells passed quality control.

#### Annotation of main cell types

Objects of different patients and samples were merged, log-normalized and integrated per patient using Harmony (v.1.2.1)^69^. Different cell types were identified looking at the expression of relevant tumour, T cell and APC marker genes on gene-weighted kernel density plots (Extended Data Fig. 3a,b). For downstream analyses, specific cell types were selected, reintegrated and reclustered.

#### Annotation and analyses within CD8^+^ T cells

The Seurat clusters expressing *CD3D* and/or *CD8A* were selected as T cells and reintegrated using Harmony. During the principal component analysis (PCA) calculation, genes related to mitochondrial function, non-coding RNA, immunoglobulins, TCR genes, stress-related genes and ribosomal genes were filtered out. Clustering was performed using the default Louvain algorithm. Seurat clusters expressing no *CD8A* and high levels of *CD4*, *ITGAX* and/or *FOXP3* were removed. Together, 28,372 CD8^+^ T cells were identified and reintegrated again (Fig. 3b). CD8^+^ Seurat clusters were then annotated using a panel of T-cell-related genes and cross-labelling with reference gene signatures from external single-cell datasets of human TILs^14,15,20,28^. Ultimately, 14 CD8^+^ T cell states were identified and annotated (Extended Data Fig. 3c). Next, CD8^+^CD39^+^ sorted single T cells of two matched patients (P8 and P15) were included in a follow-up analysis (Extended Data Fig. 10c) and processed according to the above-described pipeline. In total, 34,466 CD8^+^ T cells were annotated into 14 cell states. Notably, we observed a restructuring of exhausted T cell states. Previously annotated TCF7^+^ stem-like T_ex_ cells were largely subdivided, with one cluster retaining stem-like characteristics; another, termed CD137^high^ early T_ex_ cells, was characterized by high TNFRSF9 and XCL1/2 expression. Moreover, a new subpopulation emerged marked by expression of HSP genes. Both previously annotated natural-killer-like clusters were redistributed across multiple other clusters. CD39^−^ status was determined using each cell’s *ENTPD1* expression and the average of its ten closest neighbours to avoid false negatives due to dropouts, common in scRNA-seq.

The Gene Expression Omnibus (GEO) GSE221553 dataset^31^ was processed to extract CD8^+^ T cells, which were then annotated by label transfer, using Seurat’s functions FindTransferAnchors and MapQuery. Cells with a low predicted.celltype.score (≤0.4) were removed from subsequent analyses.

scTCR-seq data were integrated using the scRepertoire v.2.0.4 package^70^. A TCR clonotype was defined as an individual cell or group of cells with a unique paired α and β TCR sequence (the same CDR3 amino acid sequence). CD8^+^ T cells with multiple α or β TCR chains were included and considered as a unique TCR. Cells with missing α or β chains were not included in TCR analyses.

A CD8^+^ T cell cluster signature was established after differential gene expression analysis between T cells from clusters and single T cells. A MAST test^71^ was used and patient of origin was used as a latent variable. Genes with negative log_10_-adjusted *P* > 150 and expressed in >30% of cells from clusters were preselected. These preselected genes were reordered based on average log_2_-transformed fold change and the top 30 and top 100 genes were used to build the respective cluster 30 and cluster 100 signatures (Supplementary Table 4). All over-representation and gene set enrichment analyses shown were performed with fgsea v.1.28.0.

#### Annotation and analysis within tumour cells

The Seurat clusters expressing *MCAM* and *PMEL* were selected as tumour cells and anchor-based integration per patient was performed. Seurat clusters expressing *CD8A*, *CD4* and *ITGAX* were filtered out. SCTransform was performed by regressing the percentage of mitochondrial genes and gene counts, after which remaining tumour cells were reintegrated with anchor based CCA integration and reclustered. We used the tool infercnv v1.20.0 to confirm the malignant nature of selected tumour cells. APC and T cells were used as a reference (Extended Data Fig. 5a). The infercnv was run with 0.1 cut-off for minimum average read counts per gene.

Together, 25,009 tumour cells were processed. Tumour Seurat clusters were annotated based on melanoma phenotype-specific markers and on cross-labelling with reference gene signatures from external single-cell datasets of melanoma tumour cells^4,33–35^. Tumour cells were scored for each of these gene signatures using AUCell (v.1.24.0)^72^. The scores were aggregated and scaled across the Seurat clusters. Each Seurat cluster was annotated with the highest scoring phenotype. Clusters with the same annotation were combined (Extended Data Fig. 5b). We identified nine tumour cell states (Fig. 3h). The Seurat cluster defined by low gene counts was excluded from downstream analysis.

#### Analyses and annotation of APCs

The Seurat clusters expressing *ITGAX* and/or *CD19* were selected as APCs and reintegrated using Harmony. Seurat clusters expressing *PMEL*, *MCAM* or *CD8A* were removed. Together, 11,382 APCs were included and reintegrated. The resulting subset was then split across three APC types; monocytes/macrophages (7,911), DCs (2,405) and B cells/plasma cells (1,066), based on scGate (v.1.6.2)^73^ analysis. One of the Seurat clusters was reintegrated, reclustered and subdivided because it contained proliferating cells of all APC types (Fig. 3i). During PCA calculation, the same features as for CD8^+^ T cells were filtered out.

APC types were then annotated for specific cell states using a panel of APC-related genes and cross-labelled with reference gene signatures from external single-cell datasets of human TILs^31,36–42^. We identified 21 APC cell states. In follow-up analyses, only single APCs and APCs from T cell–APC clusters were taken into account. Analyses on specific APC types included only patients with at least 20 APCs in T cell clusters.

#### Cell–cell communication analysis to compare CD8^+^ T cell interactions with tumour cells or APCs

We created a curated list of ligand–receptor pairs using Nichenet’s weighted network ligand–receptor file, including only pairs with a weight of >0.75 (weighted_networks_nsga2r_final.rds). The list was further selected by including only pairs that also met one of the following criteria: (1) present in CellChat’s (CellChatDB.human.rda) curated database for annotations^74^; (2) present in CellChat protein–protein interaction experimental data (PPI.human.rda); (3) Nichenet^75^ database weight >0.9 or (4) Nichenet database weight >0.8 and present in CellTalk^76^ (human_lr_pair.txt) or SingleCellSignalR^77^ (data_LRdb.rda) curated databases. Finally, only the pairs with receptors with subcellular localizations encompassing the key terms ‘cell membrane’ or ‘surface’ in UniProtKB were considered.

Ligands and receptors that were expressed in <10% of senders or receivers in clusters were filtered out. Ligands were ranked based on their predicted activity using nichenetr (v.2.2.0)^75^. Geneset parameter was set to upregulated genes in the interacting versus non-interacting CD8^+^ T cell population (p_val_adj<0.05, avg_log2FC > 0.1 and pct.1 > 0.05). Ligand and receptors were traced back to specific cell types or states based on expression across all senders or receivers. Receptors were associated to one of the following T cell states: (1) T_ex_, merging TOX^high^ T_ex_, GZMK^high^ T_ex_, LAG3^high^ T_ex_ and TCF7^+^ stem-like T_ex_ cells; (2) T_prol_, merging MKI67^high^ T_ex_/T_prol_, MKI67^+^ T_ex_/T_prol_ and MKI67^+^ T_em_-NK like/T_p__rol_ cells; (3) T_n_/T_mem_, merging T_n_ and T_n_/T_mem_ cells; (4) T_em_, merging early T_em_, T_em_ and T_em_-NK like cells; (5) ISG^+^ and (6) Tc17 MAIT. If the average expression of a receptor in one subgroup exceeded the mean plus one s.d. of the average expressions across all subgroups, and this occurred exclusively in that subgroup, the receptor was assigned to it. If multiple subgroups or none met this threshold, the receptor was categorized as unspecific, which means it is shared between multiple or all T cell states. Ligands were associated with APCs or tumours using the same criteria, only considering cells from clusters. CD8^+^ T cells were taken into account for the average expression levels but were not accounted for in the ligand classification.

#### Cell–cell communication analysis focused on cluster-enriched tumour or APC cell states

In a second cell communication analysis, we focused on interactions between T cells and tumour cells or between T cells and APCs separately. For this, we used our previously curated database and prioritized ligands expressed in the tumour or APC cell states enriched in T cell clusters. As possible senders, we considered the tumour cells or APCs for each identified cell state. The minimum percentage for ligand expression was set at 35% in the cells from clusters at any cell state. Receivers were defined as all interacting CD8^+^ T cells (from APC or tumour clusters) and the threshold was set at 10%. Ligands were then associated to the cluster-enriched cell states if their averaged expression exceeded that of the mean plus s.d. across groups. If the condition was met exclusively in one of the cluster-enriched groups, the ligand was labelled as specific. Receptors were classified as described above. Interacting and non-interacting cells were included in the analysis. The geneset parameter was defined by comparing T cells from tumour or APC clusters to those in singlets.

### REP of TILs from patient material

Tumour digests were thawed, stained and sorted as described above. Four populations were sorted from live cells: tumour singlets, tumour–CD8^+^ T cell clusters, APC–CD8^+^ T cell clusters and CD8^+^ T cell singlets. For some experiments, single CD8^+^CD39^+^ T cells were also sorted from live cells. The research-REP (R-REP) was performed according to a protocol adjusted from a previous study^78^. In brief, sorted CD8^+^ T cell populations were plated at 100–150 cells per well in round-bottom tissue-culture-treated 96-well plates (650-180, Greiner) in 100 μl RPMI medium supplemented with 10% human serum, 5% FBS, 100 U ml^−1^ penicillin–streptomycin, 300 IU ml^−1^ IL-2, 10 ng ml^−1^ IL-7, 10 ng ml^−1^ IL-15, 0.8 μg ml^−1^ phytohemagglutinin (PHA, R30852801, Thermo Fisher Scientific) and 50,000 irradiated feeder cells. Feeder cells consisted of 45,000 35-Gray-irradiated allogeneic PBMCs (mix of two donors) and 5,000 50-Gray-irradiated EBV-JY cells. After 7 days, 100 μl of medium without PHA was added. Then, after 10–11 days, T cells were collected and rested for at least 3 days in RPMI medium supplemented with 10% FBS, 100 U ml^−1^ penicillin–streptomycin and 100 IU ml^−1^ IL-2, before functional tests were performed. For the clinical-REP (C-REP), the same populations were sorted, but cells were collected in RPMI supplemented with 20% human serum. Sorted CD8^+^ T cell populations were plated at 10,000 cells per well in flat-bottom tissue-culture-treated 24-well plates in 2 ml 20/80 AIM V/RPMI medium (AIM V, 12055083, Thermo Fisher Scientific) supplemented with 10% human serum, 100 U ml^−1^ penicillin–streptomycin, 3,000 IU ml^−1^ IL-2 and 30 ng ml^−1^ anti-hCD3 (OKT3) and 2 × 10^6^ irradiated feeder cells. Feeder cells consisted of a mix of two PBMC donors that were irradiated with 40 Gy. After 7 days, 1 ml of medium was refreshed with medium without anti-hCD3 antibodies. After 10–11 days, T cells were collected and rested for at least 3 days in 20/80 AIM V/RPMI medium with 10% human serum, 100 U ml^−1^ penicillin–streptomycin and 100 IU ml^−1^ IL-2, before functional tests were performed. Sorted melanoma tumour cells were cultured in tissue-culture-treated flat-bottom plates in DMEM or Ham’s F-10 medium (11550043, Gibco) supplemented with 10% FBS and 100 U ml^−1^ penicillin–streptomycin and adherent cells were split when reaching confluency.

### Secondary co-cultures after REP

Details on secondary co-cultures are provided in Supplementary Tables 6 and 7. To assess cytokine production, CTV-labelled CD8^+^ T cells were co-cultured with autologous melanoma tumour cells for 4 h at the indicated ratios. After 2 h 1:1,000 diluted Golgiplug (555029, BD) was added to the culture. After co-culture, an intracellular staining protocol was performed as described above and cytokine production was measured by flow cytometry. For killing assays, melanoma tumour cells were seeded into tissue-culture-treated 96-well flat-bottom plates, after which unlabelled T cells were added at the indicated ratios. At the end of co-cultures, T cells were removed from the plates and tumour cell viability was determined using CellTiter-Blue (G8081, Promega) according to the manufacturer’s instructions.

### Mouse experiments

#### ACT of primary human T cells in tumour-bearing NSG mice

Animal work procedures performed in NSG mice were approved by the animal experimental committee (Instantie voor Dierenwelzijn) of the NKI according to Dutch law and performed in accordance with ethical and procedural guidelines established by the NKI and Dutch legislation. All animals are housed in disposable cages in the laboratory animal centre (LAC) of the NKI, minimizing the risk of cross-infection, improving ergonomics and obviating the need for a robotics infrastructure for cage-washing. The mice were kept under specific-pathogen-free conditions under a controlled filtered air humidity (55–65%), temperature (21 °C) and light–dark cycle from 07:00 to 19:00. For all mouse experiments, mice were randomized into treatment groups by tumour size on the day of ACT. Randomization ensured that the treatment groups were balanced with respect to mean tumour size and s.d. at the baseline.

Primary human T cells were isolated and transduced with the MART-1-specific TCR as described above. For the experiment, a mixture of 20:80 MART-1-specific:non-specific T cells was made and this mixture was co-cultured with a MART-1-expressing BLM cell line in a tumour:T ratio of 2:1 for 4 h. After 4 h, the cells were stained for CD8, NGFR/CD146 and msTCRβ, after which all T cells (CD8^+^), T cell singlets (NGFR^−^CD146^−^CD8^+^) and CD8^+^ T cell–tumour cell clusters (NGFR/CD146^+^CD8^+^) were sorted. These populations were then expanded using the R-REP protocol described above. Then, 7 days before the end of the REP, 1 × 10^6^ MART-1-expressing BLM cells in culturex BME Type III were subcutaneously (s.c.) injected into the right flanks of NSG mice (Jax, bred at NKI). On days 7 and 9 after tumour injection, mice were intravenously injected through the tail vein with PBS (control) or 1.0 × 10^7^ T cells from the respective groups. T cells were in vivo stimulated with an intraperitoneal injection of 1 × 10^5^ U hIL-2 (Proleukin, Novartis) between days 7–11. The tumour size was monitored three times a week with callipers by measuring tumour length (*L*) and width (*W*) and calculating volume using the formula *LW*^2^/2. All experiments ended for individual mice when the tumour volume exceeded 1,500 mm^3^. Male mice were used for the experiment at an age of 10–12 weeks at the start of the experiment.

#### ACT of patient TILs in PDX-bearing hIL-2-NOG mice

Animal experiments in (hIL-2) NOG mice were conducted in conformity with EU directive 2010/63 (regional animal ethics committee of Gothenburg approvals 4684/23). All animals in Gothenburg are housed in sterile air-ventilated cages in the laboratory animal centre (EBM). The mice were kept under specific-pathogen-free conditions under controlled filtered air humidity (45–70%), temperature (19–21 °C) and a light–dark cycle from 07:00 to 19:00.

CD8^+^ T cell singlets, CD8^+^ T cell–tumour cell clusters and CD8^+^ T cell–APC clusters from a patient digest were sorted and expanded using the R-REP as described above. After REP, T cells were frozen. PDX material (passage 2), generated from the same patient material, was digested and 0.5 × 10^6^ tumour cells were s.c. injected into the flank of immunocompromised, severe combined immune deficient interleukin-2 chain receptor-γ knockout (NOG, Taconic, controls) mice or NOG mice transgenic for human IL-2 (hIL-2-NOG, Taconic, ACT groups). Tumour growth and weights of the mice were monitored twice a week throughout the experiment. Tumour growth was measured using callipers. When tumours showed consistent growth on repeated measurements (day 19 after tumour injection), TILs of the respective groups were thawed and 5 × 10^6^ TILs were intravenously injected through the tail vein into the hIL-2-NOG mice. Then, 2 weeks later (day 33 after tumour injection), all mice were euthanized due to body weight loss and material was collected for flow cytometry and immunohistochemistry analysis. Female mice were used for the experiment at an age of 6–8 weeks at the start of the experiment.

Flow cytometry was performed as described above, with a panel staining CD3, NGFR, CD146, CD137, PD1 and CD39. For immunohistochemistry, tissue from the PDX-bearing mice was fixed in 4% formalin, dehydrated and embedded in paraffin. Sections of 4 µm were mounted onto positively charged glass slides and dried overnight at 37 °C. The slides were stained using an autostainer (Autostainer Link 48, Dako). Primary antibodies were against CD3 (IR503, Dako, ready to use), CD8 (C8/144B, IR623, Dako, ready to use), CD137 (E6Z7F XP, 19541, Cell Signaling Technology, 1:250) and PD-L1 (E1L3N XP, 13684, Cell Signaling Technology, 1:200). The slides were finally counterstained with haematoxylin, dehydrated and mounted with Pertex. Stained slides were scanned using the Olympus VS200 slide scanner system. Positive cell detection of CD3^+^, CD8^+^ and CD137^+^ cells was performed in Qupath (v.0.5.1)^79^. The RGB signal was first split into two separate stains with the stain vector [0.65111 0.70119 0.29049] for hematoxylin and [0.26917 0.56824 0.77759] for DAB. The positive cell detection plugin was set to detect cells for which the DAB optical density in the whole cell was higher than 0.01. The script for automation of this workflow is available on request. To quantify PD-L1 expression, we used the pixelwise *H*-score as previously described^80^. The method was implemented in QuPath and the resulting score can range between 0 (no expression) and 300 (maximum expression).

#### ACT of patient TILs in PDX-bearing-NSG mice

The same PDX material and TILs as described in the ‘ACT of patient TILs in PDX-bearing hIL-2-NOG mice’ section was used. In total, 0.5 × 10^6^ tumour cells in culturex BME Type III were s.c. injected into the right flanks of NSG mice (Jax, bred at NKI). When tumours reached an average size of between 20 and 50mm^3^ (day 19 after injection), they were treated with 1.0 × 10^7^ thawed T cells from the respective groups. T cells were thawed 1 day before ACT. Then, 1 × 10^5^ U hIL-2 was injected intraperitoneally once daily after ACT as described before^43^. Tumour size was monitored three times a week with callipers as described in the ‘ACT of primary human T cells in tumour-bearing NSG mice’ section. All experiments ended for individual mice when the tumour volume exceeded 1,000 mm^3^. Male mice were used for the experiment at an age of 8 weeks at the start of the experiment.

### Statistics and reproducibility

Throughout the paper, different statistical tests were used as indicated in each figure legend. Two-sided tests were used unless stated otherwise. For average cell state/TCR analyses (Fig. 3b,c,h,j and Extended Data Figs. 6b and 10e), statistical significance was assessed using Bonferroni-adjusted *P* values from generalized linear mixed-effects models with a binomial distribution. For each cluster, the proportion of events was modelled using interaction status as a fixed effect and patient origin as a random effect. In Fig. 4d, statistical analysis was performed using two-way ANOVA followed by a Dunnett’s multiple-comparison test versus singlets, including all T cell–tumour cell co-culture ratios tested (visualized in Extended Data Fig. 7b). For all box plots, the box limits represent the interquartile range, the centre lines indicate the median, and the whiskers extend to the furthest point above the third quartile or below the first quartile within 1.5× the interquartile range. For in vivo experiments, the investigator measuring the tumours was blinded to the treatment. For other experiments, the investigators were not blinded. To ensure reproducibility, multiple biological and technical replicates were included. Technical replicates were generated during the same period in time and biological replicates were obtained during different moments in time. Complex bioinformatic analyses were always verified by a second researcher. Analyses were performed using GraphPad (v.10.4.1) and R (v.4.3.3).

### Reporting summary

Further information on research design is available in the Nature Portfolio Reporting Summary linked to this article.

## Online content

Any methods, additional references, Nature Portfolio reporting summaries, source data, extended data, supplementary information, acknowledgements, peer review information; details of author contributions and competing interests; and statements of data and code availability are available at 10.1038/s41586-025-09754-w.

## Supplementary information

Supplementary Tables Supplementary Tables 1–8.

Reporting Summary

## Source data

Source Data Fig. 1

Source Data Fig. 2

Source Data Fig. 3

Source Data Fig. 4

Source Data Extended Data Fig. 1

Source Data Extended Data Fig. 2

Source Data Extended Data Fig. 3

Source Data Extended Data Fig. 4

Source Data Extended Data Fig. 5

Source Data Extended Data Fig. 6

Source Data Extended Data Fig. 7

Source Data Extended Data Fig. 8

Source Data Extended Data Fig. 9

Source Data Extended Data Fig. 10

## Data Availability

Plotted data and statistical output supporting this study are provided in Supplementary Tables 1–8 and the source data. Processed scRNA-seq and scTCR-seq data are publicly available at the NCBI GEO (GSE283942). The raw scRNA-seq and TCR-seq files have been deposited at the European Genome–Phenome Archive under study accession code EGAS50000000785 and dataset ID EGAD50000001155. Owing to the privacy sensitivity of the raw data, requests for the data need to be made through https://ega.nki.nl, and will be reviewed by the NKI IRB and the principal investigator of the study. The request should include the research goal, specific names and email addresses of the people requesting access to the EGA data, privacy and governance aspects and intended use of the EGA data. Time from request to approval will take up to 2 weeks. Data are available on condition that no attempt is made to reidentify patients, the data are used for the requested goal, the data will not be transferred to a third party and are used in accordance with all applicable laws and regulations. After approval, the researcher will need to sign a common data access agreement with the NKI. We also used the UniProt database (https://www.uniprot.org); gene sets for GSEA (https://www.gsea-msigdb.org/gsea/index.jsp); human genome reference GRCh38 and human V(D)J reference (https://www.10xgenomics.com/support/software/cell-ranger/downloads); and reprocessed data from GEO (GSE221553)^31^. Moreover, we downloaded and used for downstream analyses files from Nichenet (https://github.com/saeyslab/nichenetr)^75^, SingleCellSignalR (https://github.com/SCA-IRCM/SingleCellSignalR)^77^, CellTalkDB (https://github.com/ZJUFanLab/CellTalkDB)^76^ and CellChat (https://github.com/jinworks/CellChat)^74^. Source data are provided with this paper.

## Code availability

Code is available at GitHub (https://github.com/PeeperLab/HeterotypicClustersR).
