# Supplementary material for: Tumour-reactive heterotypic CD8 T cell clusters from clinical samples
Source: Nature. 2025 Nov 19;649(8096):467–76. doi: 10.1038/s41586-025-09754-w (PMC12779571; doi:10.1038/s41586-025-09754-w)
Supplement: Supplementary file 2 — Reporting Summary [file 41586_2025_9754_MOESM2_ESM.pdf]

nature portfolio

Corresponding author(s): Daniel Peeper

Last updated by author(s): Oct 9, 2025

Reporting Summary

Nature Portfolio wishes to improve the reproducibility of the work that we publish. This form provides structure for consistency and transparency in reporting. For further information on Nature Portfolio policies, see our Editorial Policies and the Editorial Policy Checklist.

Statistics

For all statistical analyses, confirm that the following items are present in the figure legend, table legend, main text, or Methods section.

n/a	Confirmed	
☐	☒	The exact sample size (*n*) for each experimental group/condition, given as a discrete number and unit of measurement
☐	☒	A statement on whether measurements were taken from distinct samples or whether the same sample was measured repeatedly
☐	☒	The statistical test(s) used AND whether they are one- or two-sided<br>*Only common tests should be described solely by name; describe more complex techniques in the Methods section.*
☐	☒	A description of all covariates tested
☐	☒	A description of any assumptions or corrections, such as tests of normality and adjustment for multiple comparisons
☐	☒	A full description of the statistical parameters including central tendency (e.g. means) or other basic estimates (e.g. regression coefficient) AND variation (e.g. standard deviation) or associated estimates of uncertainty (e.g. confidence intervals)
☐	☒	For null hypothesis testing, the test statistic (e.g. *F*, *t*, *r*) with confidence intervals, effect sizes, degrees of freedom and *P* value noted<br>*Give P values as exact values whenever suitable.*
☒	☐	For Bayesian analysis, information on the choice of priors and Markov chain Monte Carlo settings
☒	☐	For hierarchical and complex designs, identification of the appropriate level for tests and full reporting of outcomes
☐	☒	Estimates of effect sizes (e.g. Cohen's *d*, Pearson's *r*), indicating how they were calculated

Our web collection on statistics for biologists contains articles on many of the points above.

Software and code

Policy information about availability of computer code

Data collection	Flow cytometry: FACSDiva (v8 or v9); ImageStream: INSPIRE (v200.1.681.0); Multiplex: InForm (v3.0)
Data analysis	Flow cytometry: Flowjo (v10.8.1); ImageStream: IDEAS (v6.3 or 6.4), FIJI (v2.14), CLIJ (v2.5), Cellpose (v2 or v3), IJ-Plugins toolkit (v2.3), Custom scripts (https://github.com/BioImaging-NKI/ImageStreamCombiner and https://github.com/BioImaging-NKI/ImageStreamAnalysis); Multiplex: HALO (v4.0.5107.357), Indica Labs HighPlex FL v4.2.14 analysis algorithm, Python (v3.12), Pandas (v2.2.3), scikit-learn (v1.5.2); Single cell RNA- and TCR-sequencing: Cell Ranger (v7.0.1), Seurat (v4.4.0), Harmony (v1.2.1), AUCell (v1.24.0), scRepertoire (v2.0.4), Infercnv (v1.20.0), scGate (v1.6.2), Nichenetr (v2.2.0), Fgsea (v. 1.28.0), R (v. 4.3.3), the code is deposited on Github: https://github.com/PeeperLab/HeterotypicClustersR; Immunohistochemistry: Qupath (v0.5.1); Data visualization and statistical analysis: GraphPad (v10.4.1) and R (v4.3.3)

For manuscripts utilizing custom algorithms or software that are central to the research but not yet described in published literature, software must be made available to editors and reviewers. We strongly encourage code deposition in a community repository (e.g. GitHub). See the Nature Portfolio guidelines for submitting code & software for further information.

Data

Policy information about availability of data

All manuscripts must include a data availability statement. This statement should provide the following information, where applicable:

- Accession codes, unique identifiers, or web links for publicly available datasets
- A description of any restrictions on data availability
- For clinical datasets or third party data, please ensure that the statement adheres to our policy

Plotted data and statistical output supporting this study are available in Supplementary Tables 1-8 or Source Data files. Processed scRNA- and scTCR-sequencing data is publicly available in NCBI GEO data repository GSE283942 (https://www.ncbi.nlm.nih.gov/geo/query/acc.cgi?acc=GSE283942). The raw scRNA- and TCR-seq files are deposited in the European Genome-phenome Archive under study accession code EGAS50000000785 (https://ega-archive.org/studies/EGAS50000000785) and dataset ID EGAD50000001155 (https://ega-archive.org/datasets/EGAD50000001155). Because of the privacy sensitivity of this raw data, requests for the data need to be made through https://ega.nki.nl, and will be reviewed by the NKI IRB and the Principal Investigator of the study. The request should include the research goal, specific names/e-mail addresses of the people getting access to the EGA data, privacy and governance aspects and intended use of the EGA data. Time from request to approval will take up to 2 weeks. Data is available on condition that no attempt is made to reidentify patients, data is used for the requested goal, data will not be transferred to a third party and is used in accordance with all applicable laws and regulations. After approval the researcher will need to sign a common data access agreement with the NKI. Briefly here, we also used the UniProt database (https://www.uniprot.org); genesets for GSEA (https://www.gsea-msigdb.org/gsea/index.jsp); human genome reference GRCh38 and human V(D)J reference (https://www.10xgenomics.com/support/software/cell-ranger/downloads); reprocessed data from GSE221553 (https://www.ncbi.nlm.nih.gov/geo/query/acc.cgi?acc=GSE221553) (ref. 1). In addition, we downloaded and used for downstream analyses files from: Nichenet (https://github.com/saeyslab/nichenetr) (ref. 2), SingleCellSignalR (https://github.com/SCA-IRCM/SingleCellSignalR) (ref. 3), CellTalkDB (https://github.com/ZJUFanLab/CellTalkDB) (ref. 4) and CellChat (https://github.com/jinworks/CellChat) (ref. 5).

1. Barras, D. et al. Response to tumor-infiltrating lymphocyte adoptive therapy is associated with preexisting CD8+ T-myeloid cell networks in melanoma. Sci. Immunol. 9, eadg7995 (2024).
2. Browaeys, R., Saelens, W. & Saeys, Y. NicheNet: modeling intercellular communication by linking ligands to target genes. Nat. Methods 17, 159–162 (2020).
3. Cabello-Aguilar, S. et al. SingleCellSignalR: inference of intercellular networks from single-cell transcriptomics. Nucleic Acids Res. 48, e55–e55 (2020).
4. Shao, X. et al. CellTalkDB: a manually curated database of ligand–receptor interactions in humans and mice. Brief. Bioinform. 22, bbaa269 (2021).
5. Jin, S. et al. Inference and analysis of cell-cell communication using CellChat. Nat. Commun. 12, 1088 (2021).

Research involving human participants, their data, or biological material

Policy information about studies with human participants or human data. See also policy information about sex, gender (identity/presentation), and sexual orientation and race, ethnicity and racism.

Reporting on sex and gender	Analyses based on sex and gender have not been performed.
Reporting on race, ethnicity, or other socially relevant groupings	This information has not been collected.
Population characteristics	Relevant patient characteristics have been provided in Supplementary Table 2.
Recruitment	Resected tumor material was collected from melanoma patients undergoing surgery at the Netherlands Cancer Institute/ Antoni van Leeuwenhoek Hospital (NKI-AvL) There was no specific recruitment procedure.
Ethics oversight	The study was approved by the Medical Ethical Review Board of the NKI-AvL (under studies B16MEL, IRBm23-029) and executed in compliance with the ethical regulations. All patients provided prior informed consent to use their anonymized data and tumor material for research including publication of the results in a manuscript.

Note that full information on the approval of the study protocol must also be provided in the manuscript.

Field-specific reporting

Please select the one below that is the best fit for your research. If you are not sure, read the appropriate sections before making your selection.

☒ Life sciences ☐ Behavioural & social sciences ☐ Ecological, evolutionary & environmental sciences

For a reference copy of the document with all sections, see nature.com/documents/nr-reporting-summary-flat.pdf

Life sciences study design

All studies must disclose on these points even when the disclosure is negative.

Sample size	The sample size for the in vivo experiments studying tumor volume was determined upfront using power calculation to comply with ethical guidelines.<br><br>For the PDX experiment: Comparison will be done of T cells from clusters (from tumor or APC clusters) to T cells singlets or No T cells. We

expect group 1 (control - no T cells) to reach 1500 mm3 with a SD of 500 mm3, we expect group 2-4 (T cell ACT) to reach 300-700 mm3 with a SD of 500 mm3. Using this data for G-Power analysis we would require 10 mice per group to achieve a statistical significance at an alpha of 0.05% and a power of 0.95 when using ANOVA.
BLM experiment:This experiment was set-up as a pilot experiment, with the aim to investigate the outgrowth of BLM after T cell ACT compared to control. We estimated that this amount of animals would be sufficient to determine the window. However, effect size was so big, that it was sufficient to reach statistical significance and therefore the experiment was not repeated with a bigger sample size.

For all other experiments whether the sample size was sufficient to detect the effect size above background became apparent when performing a statistical test on the results. Such tests require in general at least three biological or technical replicates.

Data exclusions

For bio-informatic APC analyses, patients were excluded if <20 cells were detected in the respective APC type from T cell clusters. For other experiments no data exclusion was performed.

Replication

To ensure reproducibility, we addressed experimental variation by including multiple biological and technical replicates. Experiments were performed in, at least, three biological replicates. All biological replicates were included in final data analyses. If biological replicates are shown, they are the average of three technical replicates unless stated otherwise. For experiments with patient material, technical replicates are shown per patient to visualize patient to patient variation. Technical replicates were generated during the same period in time and biological replicates were obtained during different moments in time. Complex bio-informatic analyses were always verified by a second researcher.

Randomization

The mice for in vivo experiments were randomized into treatment groups by tumor size on the day of ACT. The randomization was done in such a way that average tumor size and SD were similar at the start of treatment. For other experiments randomization was not applicable.

Blinding

For in vivo experiments, the investigator measuring the tumors was blinded for the treatment. For in vitro and ex vivo experiments this is not applicable, as we used well-controlled objective quantitative methods to exclude bias.

Reporting for specific materials, systems and methods

We require information from authors about some types of materials, experimental systems and methods used in many studies. Here, indicate whether each material, system or method listed is relevant to your study. If you are not sure if a list item applies to your research, read the appropriate section before selecting a response.

Materials & experimental systems

n/a	Involved in the study	
☐	☒	Antibodies
☐	☒	Eukaryotic cell lines
☒	☐	Palaeontology and archaeology
☐	☒	Animals and other organisms
☒	☐	Clinical data
☒	☐	Dual use research of concern
☒	☐	Plants

Methods

n/a	Involved in the study	
☒	☐	ChIP-seq
☐	☒	Flow cytometry
☒	☐	MRI-based neuroimaging

Antibodies

Antibodies used

PE Hamster Anti-Mouse TCRβ chain, H57-597, BD Biosciences, 553172
APC Hamster Anti-Mouse TCRβ chain, H57-597, BD Biosciences, 553174
APC Anti-human CD69, FN50, Biolegend, 310910
PE Anti-human CD69, FN50, Immunotools, 21620694X2
FITC Mouse Anti-human HLA-A2, BB7.2, BD Biosciences, 551285
BV421 Mouse Anti-human HLA-A2, BB7.2, BD Biosciences, 740082
PE Mouse Anti-human CD271 (NGFR), C40-1457, BD Biosciences, 557196
APC Anti-human CD271 (NGFR), ME20.4, Biolegend, 345108
PE Anti-human CD146, P1H12, Biolegend, 361006
APC Anti-human CD146, P1H12, Biolegend, 361016
BV421 Anti-human CD11c, Bu15, Biolegend, 337226
BUV805 Mouse Anti-Human CD8, SK1, BD Biosciences, 612889
PE Anti-human CD8, SK1, Biolegend, 344706
APC Anti-human CD8a , RPA-T8, Biolegend, 301049
FITC Anti-human CD8, SK1, Biolegend, 344704
BV711 Anti-human CD39, A1, Biolegend, 328228
PE/Cyanine5 Anti-human CD39, A1, Biolegend, 328248
APC Anti-human CD279 (PD-1), EH12.2H7, Biolegend, 329908
BV421 Anti-human CD279 (PD-1), EH12.2H7, Biolegend, 329920
CD3 FITC, SK7, BD Biosciences, 345763
FITC Mouse Anti-Human INF-γ, B27, BD Biosciences, 554700
PE Anti-human TNF-a, Mab11, Biolegend, 502909
APC Mouse Anti-human CD137, 4B4-1, BD Biosciences, 550890
PE Mouse Anti-human CD4, SK3, BD Biosciences, 566910

	BV421 Mouse Anti-human CD54 (ICAM-1), HA58, BD Biosciences, 564077<br>PE Mouse Anti-human CD58, 1C3, BD Biosciences, 555921<br>PE Mouse Anti-human HLA-ABC, G46-2.6, BD Biosciences, 560964<br>TotalSeq-C0251 anti-human Hashtag 1, LNH-94; 2M2, Biolegend, 394661<br>TotalSeq-C0252 anti-human Hashtag 2, LNH-94; 2M2, Biolegend, 394663<br>LIVE/Dead Fixable Near-IR Dead Cell Stain Kit, Invitrogen, L34976<br>CellTrace Violet Cell Proliferation Kit, Invitrogen, C34557<br>CellTrace CFSE Cell Proliferation Kit, Invitrogen, C34554<br>Anti-CD8, C8/144B, DAKO, M7103<br>Anti-CD8, C8/144B, DAKO, IR623<br>Anti-CD4, SP35, Cell Marque, 104R-16<br>Anti-CD69, EPR21814, Abcam, ab233396<br>Anti-CD11c, D3V1E, Cell Signaling, CST45581S<br>Anti-SOX10, BC34, Biocare Medical, BCARACI3099C<br>Anti-HMB45, PMEL/Melanoma gp100, Cell Signaling, 38815<br>Anti-HLA-A, EP1395Y, Abcam, ab52922<br>Anti-mouse secondary, Vector laboratories, PI-2000-1<br>Anti-rabbit secondary, Invitrogen, 31460<br>Anti-CD3, DAKO, IR503<br>Anti-CD137, E6Z7F, Cell Signaling Technology, 19541<br>Anti-PD-L1, E1L3N, Cell Signaling Technology, 13684
Validation	Antibodies were used following manufacturers instruction. All antibodies are commonly used to stain immune or tumor-related markers. Validation statements and numerous citations for all antibodies can be found on the website of the manufacturer by entering the catalog number listed above.

Eukaryotic cell lines

Policy information about cell lines and Sex and Gender in Research

Cell line source(s)	D10, FM6, BLM, A875, M063, MDA-MB-231, LCLC-103H, EBC-1, DU-145, SW480 and EBV-JY were obtained from the Peeper lab repository. Autologous primary cell lines were derived from patient tumor digest (patient information can be found in Supplementary Table 2).
Authentication	Cell lines from the Peeper lab repository were STR- profiled to confirm identity at the start of in vitro experiments.
Mycoplasma contamination	Cell lines were mycoplasma-negative at the start of in vitro experiments. Cell lines were screened for mycoplasma monthly.
Commonly misidentified lines (See ICLAC register)	No commonly misidentified cell lines were used.

Animals and other research organisms

Policy information about studies involving animals; ARRIVE guidelines recommended for reporting animal research, and Sex and Gender in Research

Laboratory animals	Experiments performed at the NKI were done with NOD-scid IL2ry-null (NSG) mice (Jax, bred at the NKI). Male mice were used for the experiment at an age of 10-12 weeks at the start of the experiment (see methods: (1) ACT of primary human T cells in tumor bearing-NSG mice) or at an age of 8 weeks at the start of the experiment (see methods: (3) ACT of patient TILs in PDX bearing-NSG mice). Experiments performed in Gothenburg were done using severe combined immune deficient interleukin-2 chain receptor γ knockout (NOG, Taconic, controls) mice or NOG mice transgenic for human interleukin-2 (hIL2-NOG, Taconic, ACT groups). Female mice were used for the experiment at an age of 6-8 weeks at the start of the experiment (see methods: (2) ACT of patient TILs in PDX bearing-hIL2-NOG mice).
Wild animals	The study did not involve wild animals.
Reporting on sex	Analyses based on sex have not been performed, as each individual animal experiment only contained one sex.
Field-collected samples	The study did not involve samples collected from the field.
Ethics oversight	Animal work procedures performed in NSG mice were approved by the animal experimental committee (Instantie voor Dierenwelzijn) of the NKI according to Dutch law and performed in accordance with ethical and procedural guidelines established by the NKI and Dutch legislation. Animal experiments in (hIL2-)NOG mice were conducted in conformity with EU directive 2010/63 (regional animal ethics committee of Gothenburg approvals #4684/23).

Note that full information on the approval of the study protocol must also be provided in the manuscript.

Flow Cytometry

Plots

Confirm that:

☒ The axis labels state the marker and fluorochrome used (e.g. CD4-FITC).

☒ The axis scales are clearly visible. Include numbers along axes only for bottom left plot of group (a 'group' is an analysis of identical markers).

☒ All plots are contour plots with outliers or pseudocolor plots.

☒ A numerical value for number of cells or percentage (with statistics) is provided.

Methodology

Sample preparation	For flow cytometry, culture medium was removed and cells were washed with 0.1% BSA in PBS. For surface staining, cells were stained with the indicated antibodies diluted in 0.1% BSA in PBS for 30 min on ice in the dark. For intracellular staining, cells were stained using the FOXP3 kit (00-5523-00, Invitrogen) according to manufacturer's instructions. A list of antibodies used can be found in Supplementary Table 8. After staining, cells were washed twice with 0.1% BSA in PBS and measured using a BD LSRFortessa, BD LSR-II SORP or BD FACSymphony A5 SORP flow cytometer with FACSDiva (v8 or v9) acquisition software. Data was analyzed using Flowjo (v10.8.1). For primary human tumor samples, previously frozen tumor digest was thawed and washed twice with RPMI, supplemented with 10% FBS and 1:1,000 benzonase nuclease (purity > 90%) (70746-3, VWR). Cells were washed an additional time with 0.1% BSA in PBS after which they were stained with antibody mix for 30 min on ice in the dark. After staining, cells were washed twice with 0.1% BSA in PBS prior to flow cytometry or sorting. When indicated, samples were washed and stained with 2% BSA in PBS and sorted in 2% FBS in PBS. Cell sorting was performed using a BD FACSAria Fusion cell sorter with an 85, 100 or 130 µM nozzle depending on the size of cells and clusters sorted. Sorted cells were collected in RPMI supplemented with 20% FBS, before proceeding to downstream processing.
Instrument	BD LSRFortessa; BD LSR-II SORP, BD FACSymphony A5 SORP, BD FACSAria Fusion
Software	Collection using FACSDiva (v8 or v9). Analysis using Flowjo (v10.8.1).
Cell population abundance	Flow cytometry was used to measure the relative abundance of a cell population of interest within a tumor sample or experimental condition. If possible, at least 30.000 cells per sample/condition were measured. Purity of sorted cell populations was checked by remeasuring the sorted population. Purity check was not done in all samples, due to the low abundance of certain sorted populations (e.g. clusters).
Gating strategy	Cells were gated based on FSC-A and SSC-A, after which live cells were identified. Additional steps in the gating can be found in Extended Data Figure 1b, Extended Data Figure 2a or Extended Data Figure 9a.

☒ Tick this box to confirm that a figure exemplifying the gating strategy is provided in the Supplementary Information.
